# Jagged1 Instructs Macrophage Differentiation in Leprosy

**DOI:** 10.1371/journal.ppat.1005808

**Published:** 2016-08-17

**Authors:** Jon Kibbie, Rosane M. B. Teles, Zhiming Wang, Patrick Hong, Dennis Montoya, Stephan Krutzik, Seung Lee, Ohyun Kwon, Robert L. Modlin, Daniel Cruz

**Affiliations:** 1 Department of Microbiology, Immunology and Molecular Genetics, University of California, Los Angeles, Los Angeles, California, United States of America; 2 Department of Medicine, Division of Dermatology, University of California, Los Angeles, Los Angeles, California, United States of America; 3 School of Petrochemical Engineering, Changzhou University, Changzhou, Jiangsu, People’s Republic of China; 4 Department of Medicine, Division of Cardiology, University of California, Los Angeles, Los Angeles, California, United States of America; 5 Department of Chemistry and Biochemistry, University of California, Los Angeles, Los Angeles, California, United States of America; University of Massachusetts Medical School, UNITED STATES

## Abstract

As circulating monocytes enter the site of disease, the local microenvironment instructs their differentiation into tissue macrophages (MΦ). To identify mechanisms that regulate MΦ differentiation, we studied human leprosy as a model, since M1-type antimicrobial MΦ predominate in lesions in the self-limited form, whereas M2-type phagocytic MΦ are characteristic of the lesions in the progressive form. Using a heterotypic co-culture model, we found that unstimulated endothelial cells (EC) trigger monocytes to become M2 MΦ. However, biochemical screens identified that IFN-γ and two families of small molecules activated EC to induce monocytes to differentiate into M1 MΦ. The gene expression profiles induced in these activated EC, when overlapped with the transcriptomes of human leprosy lesions, identified Jagged1 (JAG1) as a potential regulator of MΦ differentiation. JAG1 protein was preferentially expressed in the lesions from the self-limited form of leprosy, and localized to the vascular endothelium. The ability of activated EC to induce M1 MΦ was JAG1-dependent and the addition of JAG1 to quiescent EC facilitated monocyte differentiation into M1 MΦ with antimicrobial activity against *M*. *leprae*. Our findings indicate a potential role for the IFN-γ-JAG1 axis in instructing MΦ differentiation as part of the host defense response at the site of disease in human leprosy.

## Author Summary

Mycobacterial diseases, such as leprosy, continue to be serious causes of mortality and morbidity worldwide. They pose a unique treatment challenge due to their ability to modify the immune response in infected individuals. For example, in leprosy there are two distinct manifestations of the disease, each characterized by the immune response of the individual. One results in a more disseminated and severe form of the disease, lepromatous leprosy, and the other is a more limited form with marked antimicrobial activity, tuberculoid leprosy. These differences in the immune response can be characterized by the phenotype and activation state of the macrophage. We illustrate how the local endothelial microenvironment can “educate” macrophages, identifying Jagged1 and select small molecules that can regulate this pathway. Therefore, these studies identify a potential strategy to intervene in infection and inflammation, by targeting macrophage instruction at the site of disease. Through the integration of *in vitro* modeling and gene expression profiles at the site of disease, we found that Jagged 1 harnesses the endothelial microenvironment to instruct antimicrobial macrophage responses in leprosy.

## Introduction

When circulating monocytes enter the site of disease, local cues from the tissue microenvironment direct their differentiation into specialized MΦ equipped for diverse tasks [1–3]. While classically activated M1 MΦ with antimicrobial activity promote host defense against intracellular pathogens, alternatively activated (M2) MΦ perform homeostatic functions including phagocytosis critical to tissue remodeling [1–6]. In leprosy, the divergence of MΦ functional programs correlate with the clinical disease spectrum [7–9]. In the self-limited, tuberculoid (T-lep) form of leprosy, disease lesions contain well-organized granulomas with M1 MΦ, expressing the MΦ marker CD209, but negative for the haptoglobin receptor CD163, yet armed with antimicrobial effector function [7]. By contrast, in the progressive, lepromatous (L-lep) form of leprosy, patient lesions are characterized by disorganized granulomas containing MΦ which co-express CD209 and CD163 but lack antimicrobial activity. Instead, these MΦ are programmed with phagocytic function, which results in the accumulation of host-derived lipids and favors mycobacterial growth [10, 11], and are therefore referred to as M2 MΦ. These data raise the question regarding the mechanisms by which clues from the microenvironment influence MΦ programming at the site of infection.

As a gatekeeper to circulating monocytes that enter disease lesions, the microvasculature is poised to deliver key differentiation cues. The very cells which allow monocytes to exit the blood and enter the site of disease, i.e. EC, were shown to trigger monocyte differentiation into MΦ [12], specifically of the M2 type [13]. Therefore, unstimulated EC have the ability to instruct M2 MΦ differentiation, yet the conditions that might alter EC to instruct M1 MΦ differentiation are not known. Here, we explore how the EC-monocyte interface can influence M1 MΦ differentiation, including upregulation of antimicrobial activity, in the context of leprosy as a human disease model.

## Results

**Fig 1:**
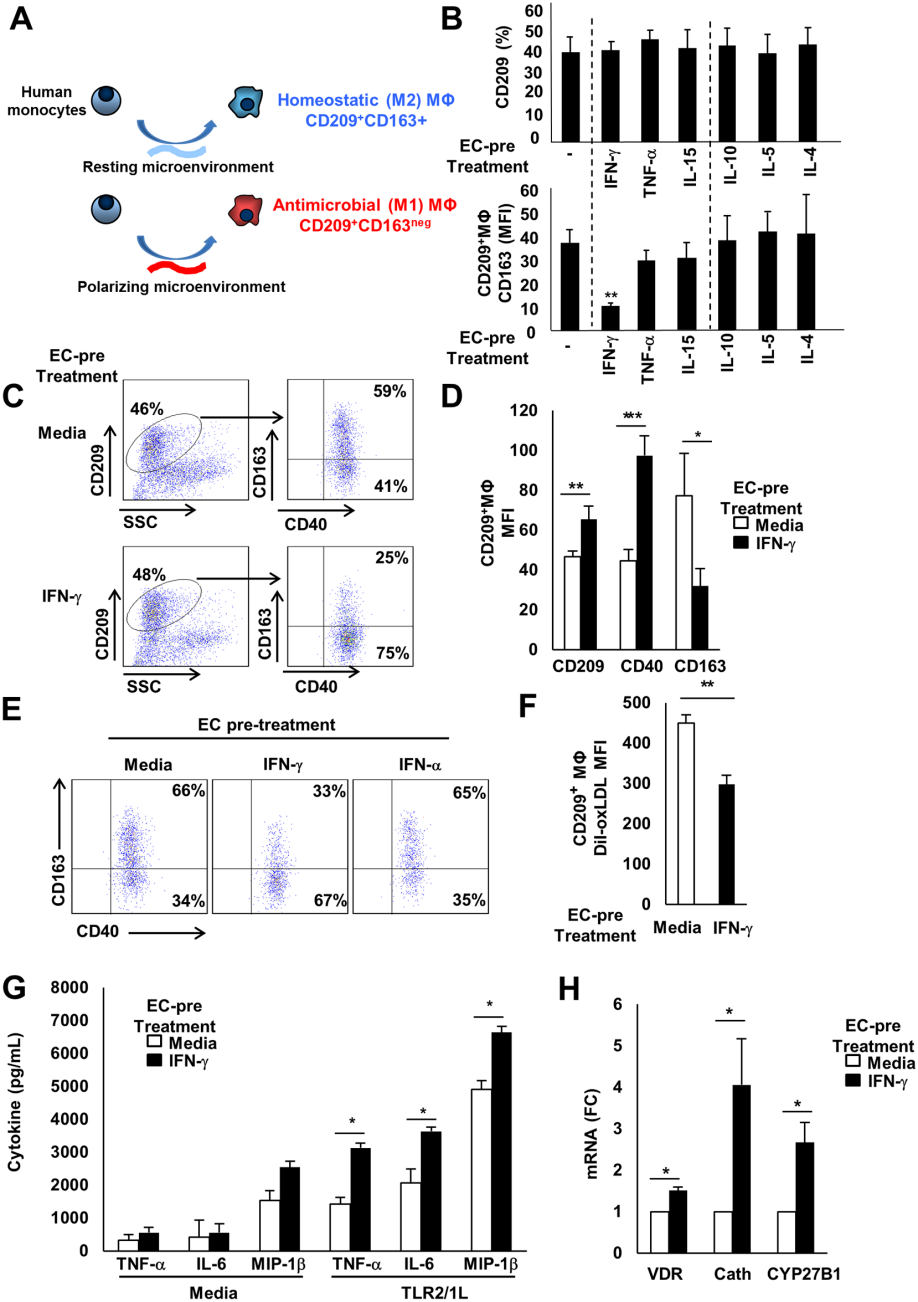
The microenvironment influences CD209+ macrophage differentiation. (A) Hypothesis: the activation state of the microenvironment instructs monocytes to differentiate into either homeostatic or antimicrobial CD209+MΦ. (B) A panel of regulatory cytokines were tested for their capacity to mediate EC-driven antimicrobial CD209+MΦ differentiation; results represent mean +/- SEM from three independent experiments performed in triplicate wells, ** p value < 0.01. (C) Representative log scale dot plots of media and IFN-γ treated ECs. (D) Compiled data comparing MΦ triggered by resting vs. IFN-γ treated EC, (n = 22 independent experiments, * p value < 0.05, ** p value < 0.01, *** p value < 0.001). (E) Representative log scale dot plots of media, IFN-γ and IFN-α treated cultures. Functional characterization of MΦ, as determined by (F) phagocytosis of Dil-oxLDL, (G) CD14+ MΦ cytokine production in response to TLR2/1L stimulation, and (H) up regulation of the vitamin D pathway in CD14+ MΦ (Cath, cathelicidin antimicrobial peptide (CAMP)). Data represent the mean +/- SEM from at least three independent donors performed in triplicate wells (* p value < 0.05, ** p value < 0.01, *** p value < 0.001).

Given the critical role the microvasculature plays in the transmigration of circulating leukocytes, it is poised to deliver instructive cues to monocytes entering the site of disease [12, 14, 15]. To investigate how the microvasculature, specifically EC, influence MΦ differentiation, we chose leprosy as a model, focusing on M1 and M2 MΦ that expressed CD209, and the relative expression of CD163, either low and high, reflecting the major MΦ phenotypes at the site of disease and endowed with distinct functional programs. We hypothesized that a resting microenvironment leads EC to instruct monocyte differentiation into M2 MΦ with phagocytic function; whereas, perturbations in the local microenvironment may direct monocytes to differentiate into M1 MΦ with antimicrobial activity (Fig 1A).

When human monocytes were cultured in the presence of several types of EC, but not vascular smooth muscle cells, we observed differentiation of monocytes into MΦ expressing CD209 (S1 Fig). As a first step to explore how the endothelial microenvironment may influence M1 vs. M2 MΦ differentiation, EC were treated with various regulatory cytokines before adding human peripheral blood mononuclear cells. After co-culture for 48h, monocyte differentiation was assessed by flow cytometry. For most of the cytokines used in the pre-treatment, EC triggered differentiation into a comparable percentage of M2 MΦ co-expressing CD209 and CD163 (Fig 1B). However, only IFN-γ-treated EC facilitated monocyte differentiation into the CD209+CD163^neg^ M1 MΦ phenotype associated with host defense [7, 16]. These M1 MΦ also expressed higher levels of CD40 [17, 18] (Fig 1C and 1D), but not the dendritic cell marker CD1a (S2 Fig). To assess whether this effect was specific to the Type II IFN, IFN-γ, we also tested the Type I IFN, IFN-α, which failed to mediate similar EC-triggered MΦ differentiation (Fig 1E). Monocyte differentiation into CD209+ MΦ was dependent on direct contact with EC and did not occur when monocytes were treated with IFN-γ in the absence of EC (S3A and S3C Fig). While the addition of IFN-γ induces very low levels of CD209 on human monocytes, direct contact with ECs elicits the highest expression of CD209 on differentiating MΦ(S3A and S3C Fig). In addition, similar results were obtained with purified CD14^+^CD16^neg^ monocytes, indicating the process was independent of lymphocytes (S3B Fig).

Next, we compared the MΦ derived from activated vs. resting EC for antimicrobial vs. phagocytic characteristics, given that our previous studies showed these programs to be divergent [7]. The M1 MΦ (CD209^+^CD163^neg^) induced by IFN-γ-treated EC, when compared to the M2 MΦ induced by resting EC (CD209^+^CD163^+^), were found to: i) take up less oxidized low density lipoprotein (oxLDL) (p <0.01) (Fig 1F), ii) produce greater amounts of pro-inflammatory cytokines in response to stimulation with a mycobacterial TLR2/1 ligand (p <0.05) (Fig 1G), and iii) express greater levels of the vitamin D antimycobacterial pathway genes Cyp27b1, VDR and cathelicidin [7] (Fig 1H). Therefore, IFN-γ, which is known to play a critical role in host defense against *M*. *leprae* and other mycobacterial pathogens [4, 8, 19, 20], licensed the EC to instruct monocyte differentiation into M1 MΦ programmed with upregulation of the vitamin D antimicrobial pathway.

### Diverse compounds can facilitate endothelial cell-driven antimicrobial macrophages

**Fig 2:**
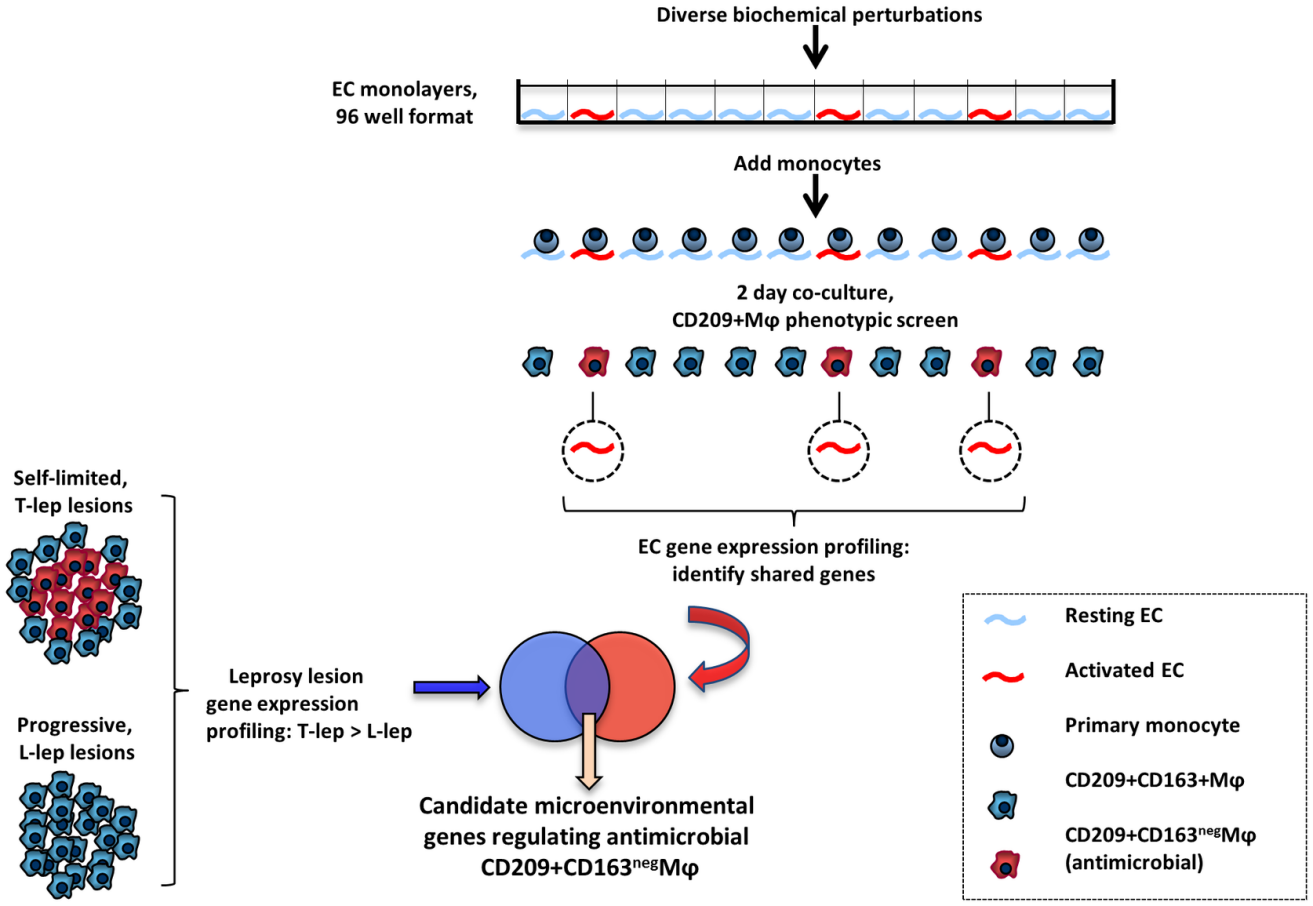
Overview of experimental design. After identification of unique activators of endothelium-directed MΦ differentiation (top), transcriptomes from activated endothelium were compared to the leprosy transcriptome to identify potential regulators of anti-microbial MΦ differentiation at the site of disease.

**Fig 3:**
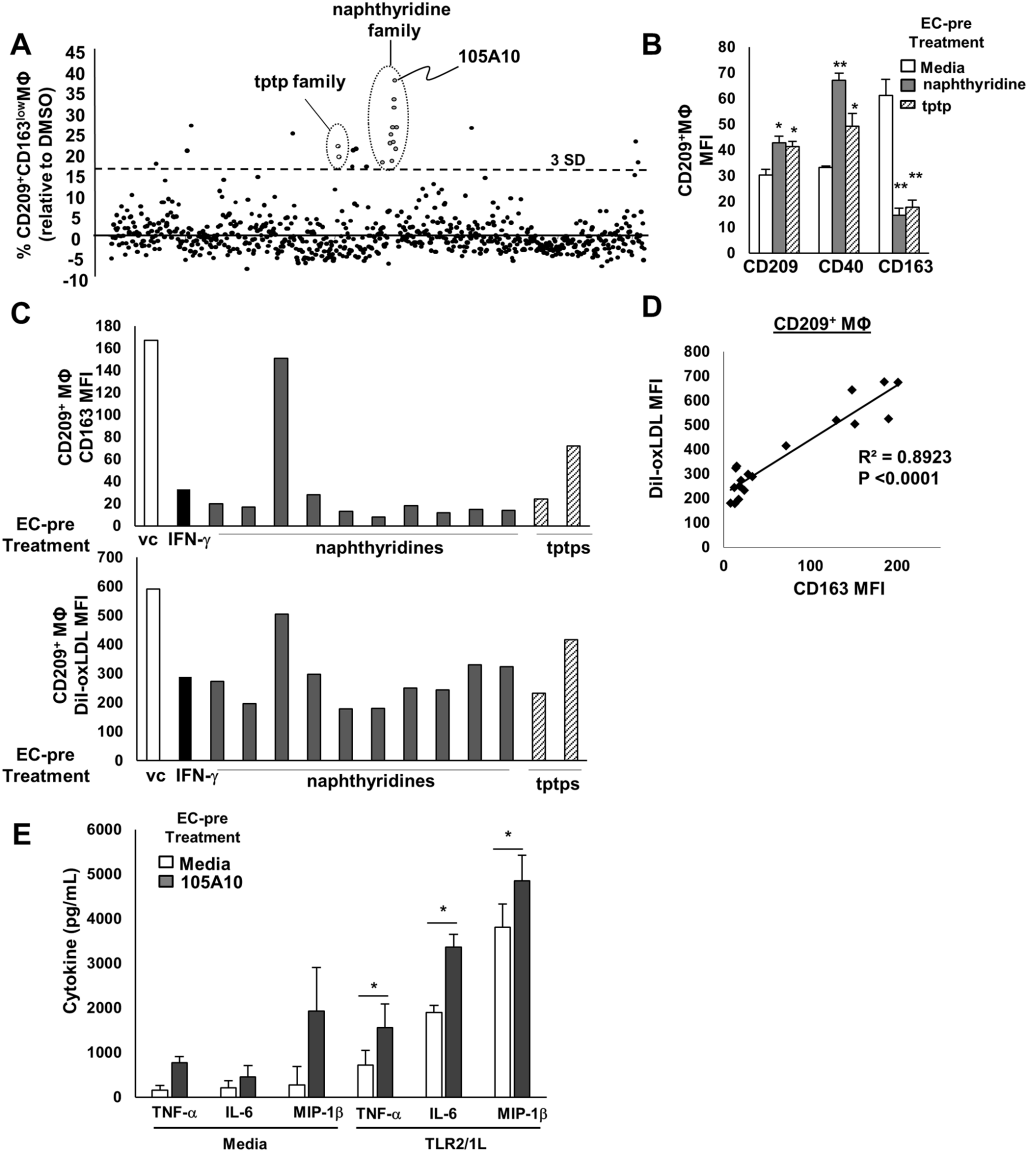
Small molecules can facilitate endothelial-driven antimicrobial MΦ. (A) Endothelial cells were treated with a small molecule library (n = 642, 10 μM) for 5 hours. EC were washed, primary mononuclear cells were added, and CD209+MΦ differentiation was assessed by flow cytometry. Polarizing compounds were selected by their capacity to drive the antimicrobial CD209+MΦ phenotype (CD209+CD163^low^ gate). Two structural families (tptp and naphthyridine) accounted for 54% of the hits, as defined by > 3 s.d. from the mean (dashed line). (B) Phenotypic validation of active tptp and naphthyridines; data represent mean and SEM of three donors (* p value < 0.05, ** p value < 0.01). (C,D) Phagocytosis of Dil-oxLDL by antimicrobial CD209+MΦ differentiated in culture with compound-treated endothelium, and correlation between CD163 expression and DiI-oxLDL uptake. (E) TLR2/1L responsiveness by CD14+MΦ derived from resting vs. naphthyridine (105A10) treated EC. Data represent the mean +/- SEM from three independent experiments performed in triplicate wells (* p value < 0.05, ** p value < 0.01).

IFN-γ is a potent inflammatory mediator that regulates an extensive gene program in EC [19–21]. We envisioned that small molecules that would facilitate EC-driven M1 MΦ differentiation may do so through convergence upon shared regulatory mechanisms (Fig 2). From a small molecule library generated by diversity-oriented synthesis (n = 642) [22], 24 compounds (3.7%) were identified which when used to treat EC, promoted M1 MΦ differentiation as measured by cell surface phenotype (Fig 3A). Two structurally distinct families, naphthyridines and tetrahydro-pyrrolo-triazolo-pyridazindiones (tptp) accounted for 13 (54%) of the “hits”, and subsequent experiments with compounds from each of these families confirmed that upon treatment of EC, they triggered differentiation of M1 MΦ (Fig 3B). As with IFN-γ, this effect was EC-dependent, since the compounds failed to directly trigger monocytes to become MΦ that express CD209 (S4 Fig). Among 81 naphthyridine analogs [22], 34 (42%) prompted EC to instruct monocyte differentiation into M1 MΦ (S5 Fig), indicating some specificity among naphthyridines. As with IFN-γ treated EC cultures, M1 MΦ derived from the compound-activated EC cultures were significantly less phagocytic than the M2 MΦ derived from the resting EC cultures (p < 0.0001) (Fig 3C and 3D). We chose the most effective compound (naphthyridine 105A10) for further analysis and found that 105A10-treated EC triggered MΦ that were also more responsive to TLR2/1 activation in terms of induction of pro-inflammatory cytokines (Fig 3E).

### Identification of candidate regulatory genes leading to the induction of antimicrobial M1 MΦ in leprosy

**Fig 4:**
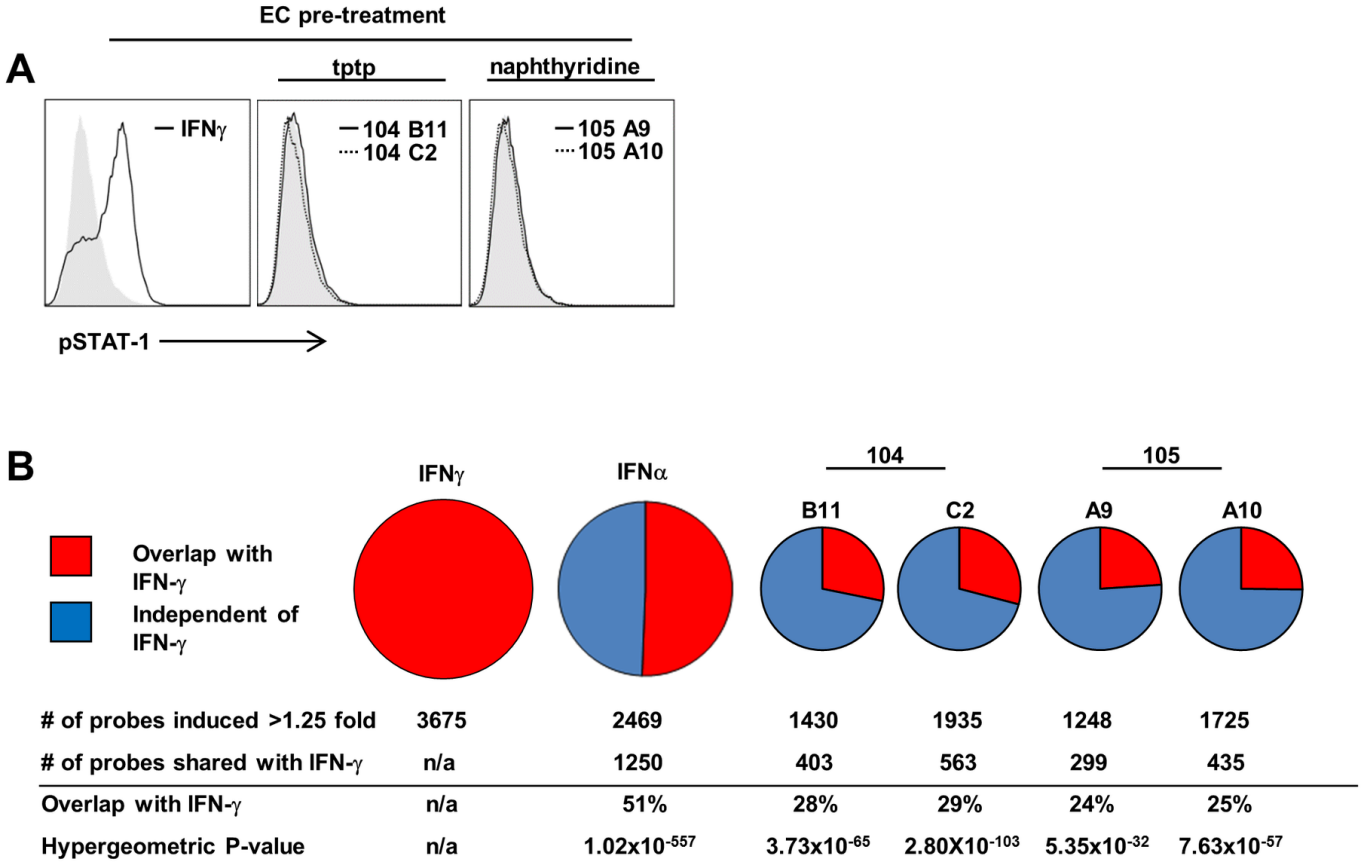
Comparative analysis of IFN-γ, IFN-α, and active small molecules. (A) Effect of IFN-γ vs. active compounds on STAT1 phosphorylation (t = 15 min) in human EC; representative of three experiments. (B) Gene expression profiling of EC treated with IFN-γ, IFN-α, 2 tptp family members (104B11, 104C2) and 2 naphthyridines (105 A9, 105A10). Number of probes (>1.25), % overlap with IFN-γ, and hypergeometric p values for over-enrichment (relative to IFN-γ) are indicated.

Having identified structurally diverse compounds that mimicked IFN-γ, we sought to use these compounds to explore the mechanisms by which EC trigger this differentiation. Since IFN-γ signaling is primarily through STAT-1, we sought to determine if active compounds from both families increased the phosphorylation of STAT-1 in treated EC. Active compounds from both tptp and naphthyridine families of compounds failed to induce phosphorylated STAT-1 (Fig 4A). To determine whether the various stimuli induce a common gene signature in EC, we measured the gene expression profiles in EC treated with either IFN-γ, IFN-α or one of four active small molecules (two naphthyridines: 105A9, 105A10 and two tptp family members: 104B11, 104C2). IFN-γ induced a broad profile (n = 3675 probes >1.25-fold induction), by comparison, the four compounds induced a more restricted profile, (range n = 1248–1935 probes >1.25-fold induction). A high proportion of the genes induced by the four compounds (24–29%) overlapped with the IFN-γ signature (hypergeometric p values for enrichment: 5.35 x 10^−32^ to 2.80 x 10^−103^, Fig 4B).

**Fig 5:**
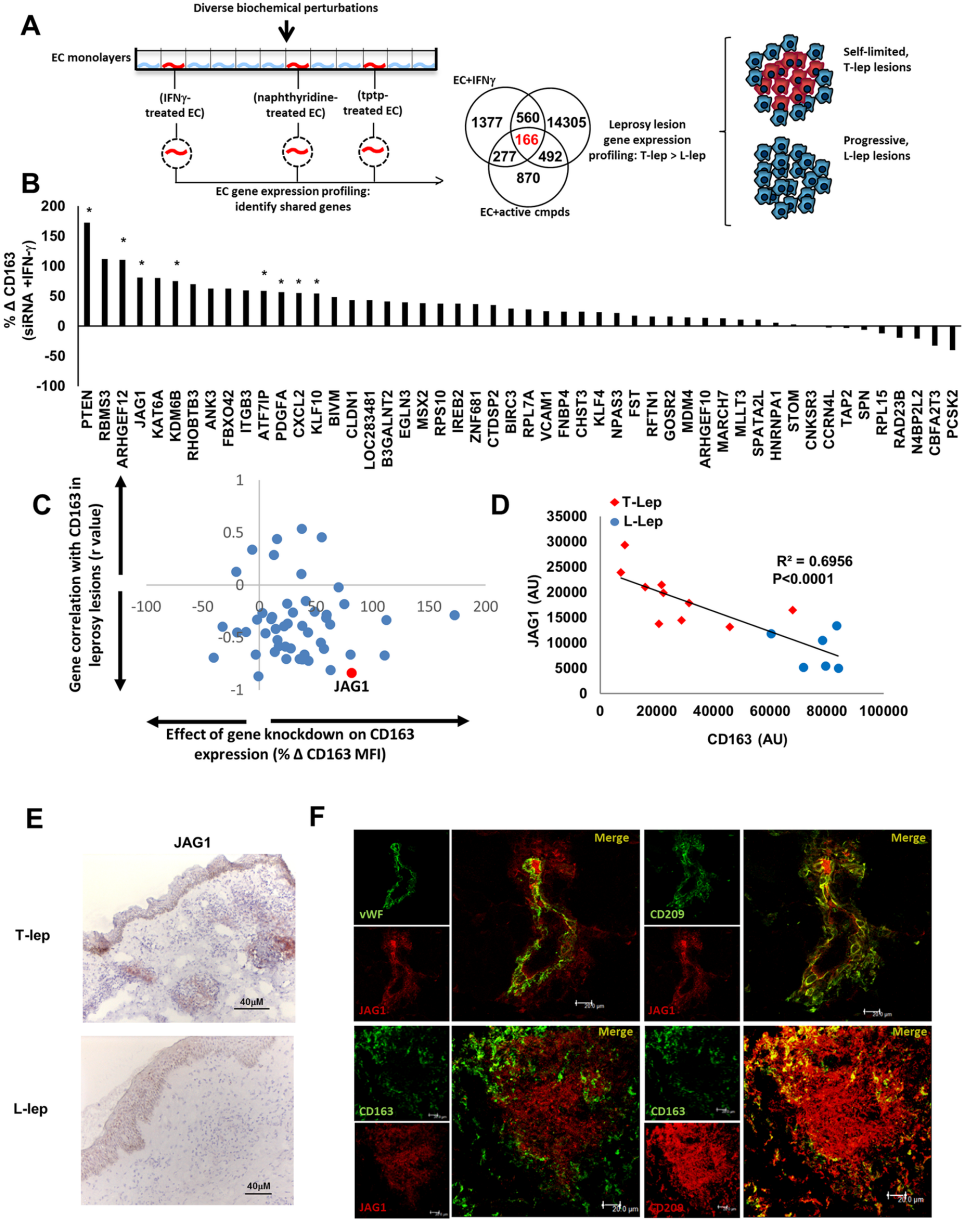
Comparative transcriptome analysis identifies JAG1 as a putative regulator anti-microbial MΦ differentiation in leprosy. (A) Genes induced in EC (FC > 1.25) by IFN-γ, naphthyridines (105 A9 and A10) and tptps (104 B11 and C2) were predicted to contain genes that also influence CD209+MΦ polarization in leprosy. Probes selectively induced (FC> 1.25) by IFN-γ and active compound treatment of the endothelium were integrated with leprosy lesions (T-lep > L-lep, FC 1.25). (B) siRNA knockdown of the top 50 candidate regulatory genes: data represent effect on IFN-γ driven effects on CD163 expression during MΦ differentiation. (* p value < 0.05) (C) The effect of siRNA-mediated candidate gene regulation was correlated with CD163 gene expression in the leprosy data set (T-Lep = 10, L-lep = 6), with JAG1 (D) emerging as a lead candidate regulatory gene. (E) Immunohistochemistry (representative of T-lep, n = 6 and L-lep, n = 4) and (F) confocal microscopy (one lesion, representative of n = 3) localizes JAG1 (IHC-red stain) expression in T-lep lesions with preferential expression in areas with CD209+CD163^neg^ MΦ and the vascular marker vWF.

To identify the genes triggered in activated EC with relevance to leprosy, we overlapped three profiles: i) induced by IFN-γ in EC, ii) induced by at least one of the four small molecules in EC; and, iii) preferentially expressed at the site of disease in the self-limited T-lep vs. the progressive L-lep form of leprosy (Figs 2 and 5A). This analysis identified 166 candidate regulatory genes, of which 50 were induced by at least two of the four compounds (S1 Table).

We next tested the role of the 50 common genes in facilitating EC-directed M1 MΦ differentiation. EC were transfected with siRNA against each of these candidate genes, and then treated with IFN-γ to induce the M1 polarizing microenvironment, followed by co-culture with primary human PBMC. In this context, monocyte differentiation into CD163+ MΦ would reflect that the M1 MΦ-polarizing effect of IFN-γ treated EC was being inhibited by the siRNA. Across five separate experiments, eight genes significantly inhibited the effect that IFN-γ exerts on the EC-driven M1 MΦ phenotype (Fig 5B, S6 Fig). In parallel, the gene expression profiles of the 50 common genes were examined in leprosy lesions, with the premise that inverse correlation with CD163 expression may indicate a role in regulating M1 MΦ differentiation at the site of disease. Among the top eight candidate genes, JAG1 demonstrated the strongest inverse correlation with CD163 expression across the spectrum of leprosy lesions (r = -0.834, R^2^ = 0.6956, P<0.0001, T-lep lesions n = 10, L-lep lesions n = 6), with greater expression in T-lep vs. L-lep lesions (fold change 2.2, p<0.0002) (Fig 5B and 5C).

After confirming that JAG1 is induced on EC following stimulation with IFN-γ (S7 Fig), we then assessed JAG1 expression at the site of disease. JAG1 expression in leprosy lesions was validated by immunohistochemistry, which demonstrated that JAG1 was expressed within the dermis and the granulomas in T-lep, but not L-lep lesions (Fig 5E, S8 Fig). We also noted perivascular labeling of JAG1 in proximity to CD209^+^ MΦ (Fig 5F), as well JAG1 expression in the microanatomic locations in which M1 MΦ (CD209^+^CD163^neg^) were found (Fig 5F). In addition, there appeared to be JAG1 staining in the epidermis of both the T-lep and L-lep lesions which is consistent with the known role of JAG1 in keratinocyte differentiation and maturation [23, 24]. Blinded analysis of JAG1 immunohistochemical staining determined a significant (p = 0.0063) increased positive staining in T-lep sections, scores ranged from 0 (absent) to 4 (highly positive). Together, these data indicated that JAG1 expression correlated with M1 MΦ accumulation at the site of disease in leprosy.

### JAG1 triggers antimicrobial macrophages in an endothelial cell-dependent manner

**Fig 6:**
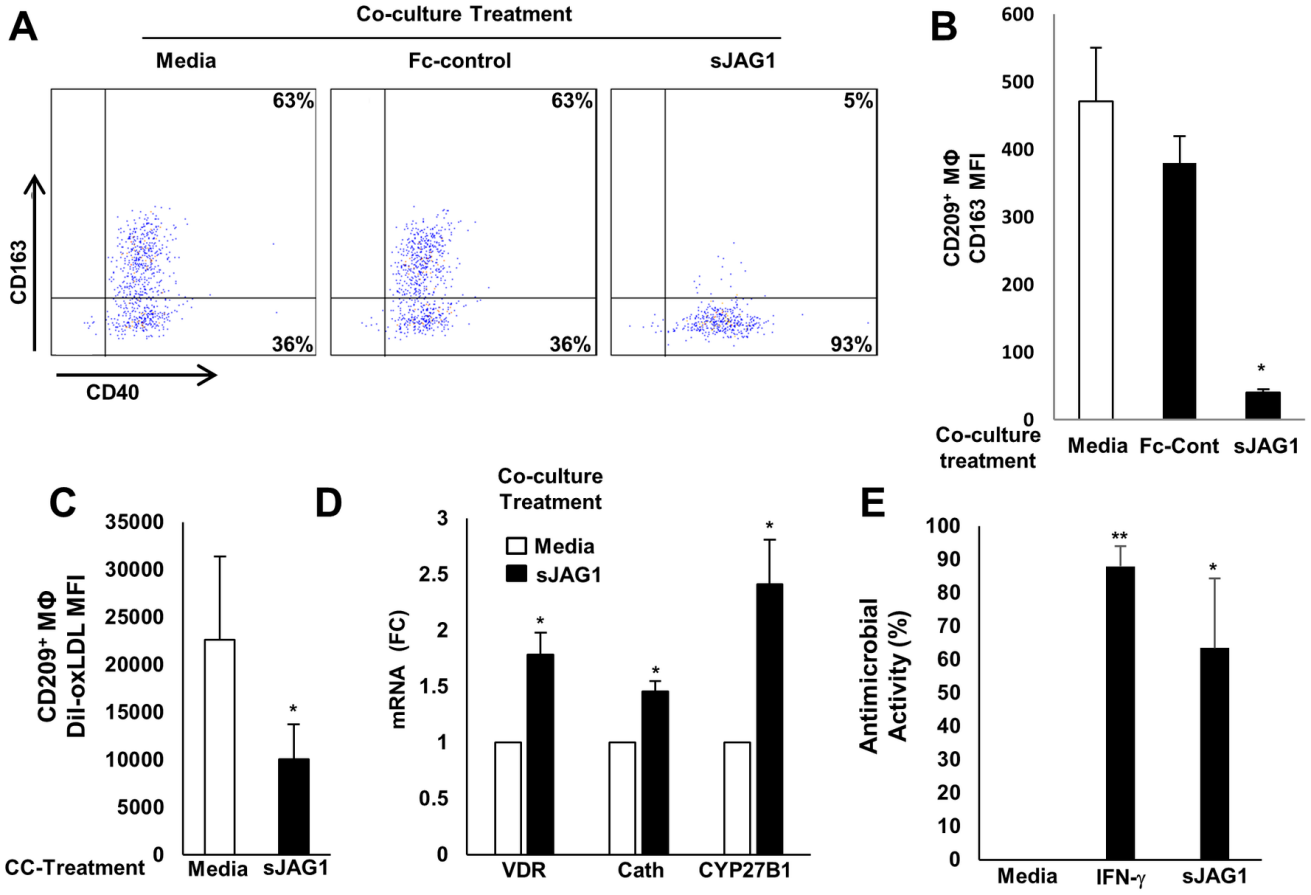
JAG1 facilitates antimicrobial CD209+MΦ differentiation. (A, B) JAG1 (250ng/ml) added to EC/primary mononuclear cell co-cultures (CC) promotes the antimicrobial CD209+MΦ phenotype (representative log scale dot plots, and mean +/- SEM of n = 6 experiments, * p value < 0.05). Functional characterization of MΦ as determined by (C) phagocytosis of Dil-oxLDL, (D) induction of the vitamin D pathway in CD14+MΦ and (E) antimicrobial ability. For antimicrobial ability determination, co-culture derived CD14+MΦ were infected with *M*. *leprae* (MOI 10:1) overnight. After infection, cells were treated with IFN-γ and supplemented with 25-D3 for a period of five days. Determination of *M*. *leprae* viability was calculated using the ratio of bacterial 16S RNA and DNA (RLEP) as quantified by qPCR. Data represent the mean +/- SEM from at least three independent donors (* p value < 0.05, * * p value < 0.01).

We next investigated whether JAG1 could instruct the differentiation of monocytes into M1 MΦ with antimicrobial function. We found that soluble JAG1 (sJAG1) facilitated EC-driven M1 MΦ differentiation (Fig 6A and 6B). Furthermore, overexpression of JAG1 in EC, as well as addition of a JAG1 agonist peptide to the co-cultures, induced the differentiation of monocytes into the M1 MΦ phenotype (S9 Fig). In contrast, addition of sJAG1 to monocytes alone did not induce MΦ differentiation (S10 Fig). Given that JAG1 is known to activate Notch 1 signaling, we determined whether Notch-downstream genes were upregulated by the addition of JAG1 to the EC/monocyte co-cultures. In comparison to untreated EC, the addition of JAG1 led to the mRNA upregulation of three prototypic Notch-downstream genes in MΦ, HES1, SOCS3 and RBPJ (S11 Fig).

Differentiation of monocytes in the presence of sJAG1 and EC yielded M1 MΦ with decreased phagocytosis (Fig 6C) and heightened induction of vitamin D-dependent antimicrobial pathway genes (Fig 6D). To determine whether EC treated by either IFN-γ or JAG1 induced differentiation of monocytes into MΦ with antimicrobial activity, MΦ differentiated in the presence of treated EC were infected with live *M*. *leprae*, and the antimicrobial response measured according to the ratio of *M*. *leprae* RNA to DNA [25, 26] (Fig 6E). As compared to MΦ differentiated in the presence of resting EC (i.e. treated with media), the MΦ induced by culture with EC treated with either IFN-γ or sJAG1 showed significant antimicrobial activity. Therefore, when monocytes encounter JAG1 in the context of EC, a differentiation program is triggered, resulting in M1 MΦ, defined by a CD209^+^CD163^neg^ phenotype and antimicrobial function. The presence of IFN-γ, JAG1-expressing EC and CD209^+^CD163^neg^ MΦ in the self-limited form of leprosy suggests that the IFN-γ-JAG1-antimicrobial MΦ differentiation pathway contributes to host defense at the site of disease in leprosy.

## Discussion

Our understanding of MΦ immunobiology has been significantly advanced through understanding of the pathways by which microbial ligands and/or cytokines program monocytes to differentiate into M1 and M2 MΦ [27, 28]. However, it is not clear how local tissue signals can differentially program the MΦ response. Signals from endothelium are involved; this default pathway triggers M2 MΦ differentiation [13]. However, the mechanisms by which monocytes, upon entering the site of disease via the endothelium, are instructed to differentiate into M1 MΦ remain elusive [1–3]. Here, we hypothesized that if EC were to encounter the proper signals, the EC microenvironment would instruct monocytes to differentiate into M1 MΦ, equipped for host defense against intracellular pathogens at the site of disease. By studying leprosy as a model, we provide evidence that upregulation of JAG1 on endothelium instructs monocytes to differentiate into M1 MΦ with antimicrobial activity.

Our data indicate that the induction of JAG1 is involved in EC instruction of M1 MΦ differentiation. In addition, the concomitant induction of Notch 1-downstream genes including HES1, SOCS3 and RBP-J mRNAs was detected in the differentiated M1 MΦ. These findings are consistent with the known ability of JAG1 to signal via Notch 1 receptors [29], and with reports that Notch 1 signaling, via SOCS3 and RBP-J [30–32] through reprogramming of mitochondrial metabolism [33], contributes to M1 MΦ differentiation. Nevertheless, since JAG1 is known to signal via several distinct receptors [34, 35], further work is necessary to identify the physiologically relevant interactions responsible for EC-driven M1 MΦ differentiation. Not only does IFN-γ induce JAG1 on EC which can influence monocyte differentiation, IFN-γ also augments TLR-induced regulation of JAG1 expression in differentiated MΦ [36]. Further studies will be required to elucidate how JAG1 can contribute to MΦ differentiation, plasticity, function and proliferation at the site of disease [37].

In addition to the role of JAG1 in regulating innate immune responses via MΦ differentiation, evidence suggests a role for JAG1 in regulating adaptive T cell responses. Patients with Alagille syndrome, in which JAG1 mutations result in a multisystem disorder [34, 38], can exhibit altered Th1 responses [35], implicating JAG1 induced signaling in T cell differentiation. *In vitro* studies have also shown that JAG1 expression on keratinocytes promotes dendritic cell maturation, which could also influence T cell responses [39]. Therefore, the expression of JAG1 by resident cells in tissue can influence both innate and adaptive immune responses.

Under resting conditions, EC instruct monocytes to differentiate in M2 MΦ [13]. M2 MΦ are highly phagocytic, and are involved in clearing various biomolecules relevant for tissue repair, removal of excess metabolic products as well as clearance of debris. However, in the context of *M*. *leprae* infection, M2 MΦ can phagocytize the bacteria, but are unable to mount an antimicrobial response. Furthermore, these M2 MΦ take up host-derived lipids, providing necessary nutrients for mycobacterial growth [40]. Therefore, the induction of M1 MΦ is required for host defense against this intracellular pathogen, as these MΦ are weakly phagocytic but exhibit a strong antimicrobial response. One direct signal at the site of infection is production of IL-15, which directly triggers M1 MΦ differentiation. In addition, our data demonstrates that IFN-γ induces JAG1 expression on EC, which also facilitates differentiation of monocytes into M1 MΦ. In the self-limited form of leprosy, JAG1 expression is restricted to microanatomical regions of the granuloma enriched for M1 MΦ. Therefore, our findings support the concept that the IFN-γ-JAG1 axis is involved in the EC instruction of the antimicrobial MΦ response against *M*. *leprae* at the site of infection.

The ability to model how the microenvironment influences the immune response at the site of disease has become feasible because of advances in analyzing increasingly complex systems. We used a cell co-culture system in which we integrated small molecule screening with gene expression profiles to look for recurrent motifs in gene activation patterns associated with EC-triggering M1 MΦ. Since none of the molecular signals we identified recapitulate the antimicrobial MΦ phenotype on their own, our findings indicate that the emergent properties inherent to more complex heterotypic systems allowed for their discovery [41]. As such, this approach provides a strategy to identify potential drugs or biologic agents that would otherwise not be identified in experiments exploring direct effects on monocyte differentiation into antimicrobial MΦ. The identification of JAG1 and other small molecules that can harness the local microenvironment to augment innate immune responses at the site of disease may hold promise for combating intracellular pathogens.

## Materials and Methods

### Reagents

IFN-γ and IL-4 (Peprotech) were used at 10ng/ml. IFN-α (PBL Interferon Source) was used at 10ng/ml. IL-15 (25ng/ml), IL-10 (10ng/ml), IL-5 (10ng/ml), fc-JAG1 (250ng/ml) and fc-control (250 ng/ml) were purchased from R&D Systems. JAG1 protein active peptide fragment (1μM) was purchased from Phoenix Pharmaceuticals. Small molecule compound libraries and analogs were synthesized in the Ohyun Kwon laboratory. Compounds were dissolved in DMSO and used at a final concentration of 10 μM.

### Co-culture experiments

Co-culture experiments were carried out as previously described [22]. In short, Primary human endothelial cells (EC) were plated to confluence in a 96 well plate. After adherence, endothelial cells were activated by indicated treatments for a period of 5 hours and subsequently washed 2–3 times to ensure removal of activation treatment. We then added human peripheral blood mononuclear cells (PBMC) at a ratio of 3 PBMC to 1 EC. Cultures were incubated at 37°C and 7% CO_2_ for a period of 48hrs. Human Umbilical Vein Endothelial Cells (HUVEC) were purchased from Lonza and used from passages 4–8. Peripheral blood mononuclear cells were isolated from healthy donors (UCLA Institutional Review Board # 92-10-591-31) using Hypaque Ficoll gradients (GE Healthcare).

### Clinical samples

Samples were retrieved by skin biopsy from patients with leprosy. The designation of tuberculoid leprosy (T-lep) and lepromatous leprosy (L-lep) was determined according to the criteria of Ridley and Jopling. Patient skin biopsies were performed at the time of diagnosis and subsequently embedded in OCT medium (Ames, Elkhart, IN), snap frozen in liquid nitrogen and stored at -80°C (24).

### Determination of STAT-1 phosphorylation

HUVEC were stimulated with IFN-γ and compounds (104 B11, 104 C2, 105 A9 and 105 A10) for 15 minutes and then stained according to manufacturer’s protocol for phosphorylated STAT-1. (N = 3)

### Cell labeling and cytokine bead array (CBA)

Cells were harvested after 48 hours incubation at 37°Celsius in 7%CO_2_. Surface expression of protein was determined using specific antibodies: CD209 (Becton Dickinson), CD40 (Becton Dickinson), CD1a (Becton Dickinson), CD163 (R&D systems), Jagged1 (R&D systems), CD14 (Becton Dickinson) and IgG controls (Becton Dickinson). Phosphorylated STAT-1 levels were determined using Anti-Human phospho-STAT1 (eBiosciences). Cytometric Bead Arrays (CBA) were used to characterize TLR2/1R activated CD14^+^MΦ supernatants. CBAs were performed on 50μL of supernatant that was harvested after 24 hours of incubation. Supernatants were tested for the presence of MIP1-β, IL-6 and TNF-α. CBA Flex kits were obtained from Becton Dickinson and performed according to manufacturer’s recommendations. Samples were acquired using *FacsCalibur* and *FacsVerse* flow cytometers and FCS files were analyzed using FlowJo software.

### MΦ TLR activation cytokine response

PBMC/EC Co-cultures were harvested after 48 hours of incubation and CD14^+^MΦ were subsequently purified using a CD14 positive selection bead assay (Miltenyi Biotec) (purity > 95%). CD14^+^MΦ from each condition (DMSO, IFN-γ and 105A10) were plated in equal number in 96 well flat bottom plates and stimulated with 10μg/ml TLR2/1 ligand (EMC Microcollections). After 24 hours of stimulation supernatants were harvested and characterized by CBA for production of MIP1-β, TNF-α and IL-6.

### Real time PCR

cDNA was generated using iScript cDNA synthesis reagent (Biorad) following manufacturers guidelines. Primers (IDT) were used for determining mRNA expression of *CYP27b*, *CAMP*, *VDR*, *and JAG1*. SYBR Green PCR Master Mix (BioRad) was used for Real Time PCR reactions and data was normalized to h36B4 gene expression (IDT). Expression values were calculated as previously described [7].

### Vitamin D pathway regulation

CD14+MΦ from co-cultures were harvested and purified as previously mentioned. After purification, MΦ were plated in 10% FCS with 25-D_3_ (10^−8^ M) (Biomol) and incubated for 24 hrs. Cells were then harvested and analyzed for *CAMP*, *VDR* and *Cyp27b1* gene expression by qPCR.

### Antimicrobial assays

Viable bacteria stocks of *M*. *leprae* were obtained from Dr. James L. Krahenbuhl of the National Hansen's Disease Programs, Health Resources Service Administration, Baton Rouge, LA. For antimicrobial assays, Endothelial/PBMC co-cultures were set up as previously mentioned. After 48 hours of incubation CD14^+^ MΦ (>90% CD209^+^) were isolated from co-cultures for infection with *M*. *leprae*. Co-culture conditioned (Media, IFN-γ and sJAG1) CD14^+^ MΦ were cultured in RPMI with 10% FCS (Omega Scientific) in the presence of live *M*. *leprae* (MOI 10:1). Infected cells were subsequently stimulated with IFN-γ (10ng/ml) in the presence of 25-D_3_ (10^−8^ M) after 24 hours of infection. To measure antimicrobial activity in *M*. *leprae*-infected MΦ (5 days post infection) we followed the protocol as previously described [40, 42]. In short, qPCR was performed to determine levels of bacterial 16S rRNA and genomic element DNA (RLEP). Expression levels of h36B4 were also evaluated to determine infectivity between all the conditions. The *M*. *leprae* 16S rRNA and RLEP primers used were as previously described [25, 26].

### Phagocytosis and *M*. *leprae* uptake assay

DiI (1,1′-dioctadecyl-3,3,3′,3′-tetramethylindocarbocyanine perchlorate)-labeled CuSO4-oxidized low density lipoprotein (Dil-Ox-LDL) from Intracel was added to co-cultures after 44 hours and further cultured for 4 hours in the presence of Dil-OxLDL to allow for uptake (50μg/ml). After incubation, Dil-OxLDL levels were determined within the CD209+ population of our stained cultures. To determine *M*. *leprae* uptake (S12 Fig), CD14^+^ MΦ were harvested from co-cultures as previously mentioned and infected with labeled *M*. *leprae*. After 24 hours of infection MΦ were harvested and stained for CD209, CD14 and CD163 expression.

### IHC and confocal microscopy

Immunoperoxidase and immunofluorescence labeling were carried out on frozen patient tissue sections. For immunoperoxidase staining, samples were initially blocked with normal horse or goat serum prior to labeling with monoclonal antibodies (JAG1 (Abcam), vWF (AbD Serotec), CD163 (AbD Serotec) and appropriate isotype controls). Sections were then labeled with biotinylated horse anti-mouse IgG or biotinylated goat anti-rabbit IgG. After labeling, sections were counterstained with hematoxylin and visualized using the ABC Elite system (Vector Laboratories). In order to determine protein co-localization in tissue sections, two-color immunofluorescence and confocal microscopy were performed. For Immunofluorescence, sections were labeled with rabbit anti-human JAG1, anti-CD163 (IgG1), anti-CD209 (IgG2b), anti-vWF (IgG1) and appropriate isotype controls. Subsequently samples were labeled with isotype-specific, fluorochrome (A488 or A568)-labeled goat anti-mouse/rabbit immunoglobulin antibodies (Molecular Probes). Nuclei were stained with DAPI (4',6'-diamidino-2-phenylindole). Double immunofluorescence of skin sections was examined using a Leica-TCS-SP MP inverted single confocal laser scanning and a two-photon laser microscope (Leica, Heidelberg, Germany) at the Advanced Microscopy/Spectroscopy Laboratory Macro-Scale Imaging Laboratory, California NanoSystems Institute, University of California at Los Angeles [26]. Blinded review of IHC samples was carried out and positive staining was scored on the scale of 0 (absent) to 4 (highest staining) relative to isotype controls. Fishers exact test was used to determine significance.

### Transfections

siRNA transfections were carried out on 2x10^4^ HUVEC in 96 well plates and 7x10^3^ HUVEC in 384 well plates. siRNA for candidate genes, siControl and siGlow were obtained from Dharmacon as was the transfection reagent Dharmafect 4. siRNA transfections were performed according to manufacturer’s recommendations using 100nM concentration of siRNA. Decrease in message in transfected cells was confirmed by qPCR and protein expression. Ectopic expression cassettes for JAG1, GFP and M11-empty vector were obtained from Genecopoeia. Plasmid transfections were carried out on HUVEC that were grown to 80–90% confluence. HUVEC were harvested and transfected with 1μg DNA using the AMAXA transfection device and HUVEC Nucleofect kit (Lonza). To determine transfection efficiency, control cells were characterized for GFP production. In addition, surface expression of transfected JAG1 was confirmed by flow cytometry.

### Microarrays

For microarrays performed on compound and cytokine treated HUVEC, ECs were seeded in 6 well plates at 1X10^6^ cells/well. Single wells were stimulated for five hours with DMSO, IFN-γ, IFN-α and compounds 104B11, 104C2, 105A9 and 105A10 at concentrations noted earlier. After incubation, mRNA was harvested using Trizol (Invitrogen), followed by RNeasy Minelute Cleanup Kit (Qiagen). mRNA samples for all arrays were processed using the Affymetrix Human U133 plus 2 platform and analyzed as previously described [22].

### Statistical analysis

Statistical significance (<.05) of experimental values was calculated using a paired two-tailed Student’s t-test. Hypergeometric p values were calculated using the online resource (http://systems.crump.ucla.edu/hypergeometric) (Tom Graeber laboratory, UCLA).

### Ethics statement

Patient samples were obtained with approval from the IRB of the University of California Los Angeles, the Institutional Ethics Committee of Oswald Cruz Foundation and the University of Southern California School of Medicine. All subjects were legal adults and provided written informed consent before participating in the study [26].

## Supporting Information

S1 Fig Contact with vascular endothelium uniquely drives CD209+MΦ differentiation. (A) Contact with vascular endothelium and not smooth muscle cells facilitates monocyte differentiation into CD209+MΦ. Histograms are representative of three independent experiments performed in triplicate.
(TIF)

S2 Fig CD1a expression on EC-driven CD209^+^MΦ subsets. Log scale dot plots are representative of more than three independent donors performed in triplicate.
(TIF)

S3 Fig Characterization of EC-driven antimicrobial CD209^+^MΦ differentiation. (A) Transwell experiments. EC were cultured in the upper chamber and treated with IFN-γ, with primary monocytes in the lower chamber. Histograms are representative of three independent donors performed in triplicate (B) IFN-γ treatment of HUVEC triggers purified CD14^+^ monocyte differentiation into antimicrobial CD209^+^MΦ. Data represent the mean +/- SEM from at least three independent donors (* p value < 0.05). (C) Treatment of PBMC with IFN-γ did not promote CD209^+^MΦ differentiation. Histograms are representative of more than three independent donors performed in triplicate.
(TIF)

S4 Fig Direct stimulation of monocytes with active compounds does not trigger antimicrobial CD209^+^MΦ differentiation. Stimulation of PBMC with active compounds (105 A9-A10, 104 B11-C2) did not result in the differentiation of CD209+MΦ. Histograms are representative of more than three independent donors performed in triplicate.
(TIF)

S5 Fig Differential effects of naphthyridine analogs in EC-driven antimicrobial CD209^+^MΦ differentiation. EC were treated with either media (m), IFN-γ (γ), 105A10, or naphthyridine analogs followed by co-culture with peripheral blood mononuclear cells. Data represent the mean +/- SEM from at least three independent donors.
(TIF)

S6 Fig siRNA screen of candidate genes involved in EC facilitated antimicrobial CD209+MΦ differentiation. (A) Data represent the mean +/- SEM from five independent donors. (B) Validation of JAG1 mRNA knockdown by qPCR. Data represent the mean +/- SEM from at least three independent experiments.
(TIF)

S7 Fig JAG1 expression on activated EC. EC were cultured with or without IFN-γ and then assessed for JAG1 expression by flow cytometry. Data represent the mean +/- SEM from two independent experiments; filled histogram represents staining observed with isotype control.
(TIF)

S8 Fig JAG1 expression in T-Lep and L-Lep biopsies. Immunohistochemistry (T-lep, n = 3 and L-lep, n = 3) staining of JAG1, vWF and IgG isotype controls. Positive staining of target proteins (JAG1 or vWF) is represented by red staining.
(TIF)

S9 Fig Stimulation of EC-monocyte co-cultures with multiple forms of Jag1. (A) Ectopic expression of JAG1 in transfected EC facilitates antimicrobial CD209^+^MΦ differentiation. Data represent the mean +/- SEM from at least three independent donors (* p value < 0.05) **(B)** Surface expression of JAG1 on M11-JAG1 transfected HUVEC. Histogram is representative of at least three independent experiments. **(C)** Addition of either JAG1 active peptide or soluble JAG1-fc protein to EC/PBMC co-culture facilitates CD209+CD163^neg^MΦ differentiation. Data represent the mean +/- SEM from at least three independent donors. (* p value < 0.05). **(D)** Dose titration of soluble JAG1-fc active protein to EC/PBMC co-culture. Data represent the mean CD163MFI on CD209+ MΦ (+/- SEM from at least two independent donors. (* p value < 0.05)).
(TIF)

S10 Fig Stimulation of monocytes with sJAG1. Direct stimulation of PBMC does not induce the differentiation of CD209^+^MΦ. Histograms are representative of more than three independent donors performed in triplicate.
(TIF)

S11 Fig Induction of NOTCH1 target genes in M1 MΦ. EC-monocyte co-cultures were treated with (black bars) or without (white bars) sJAG1 and after 2 days. NOTCH1 target gene expression was quantified by qPCR (p values: 0.02, 0.01, and 0.006 for HES1, SOCS3 and RBPJ, respectively).
(TIF)

S12 Fig Labeled *M leprae* uptake experiments. To determine uptake of *M leprae* during killing assays we used labeled *M leprea* (PE) and determined percent of CD209+ macrophages that had taken up *M leprea* by flow cytometry. Data represents three independent donors performed in triplicate. (* p value < 0.05).
(TIF)

S1 Table Candidate genes used in siRNA screen. Top fifty candidate genes used in siRNA screen. Fold change of gene expression in treated over media control HUVEC samples from microarray assays. In addition, the fold change of gene expression in T lep vs L lep tissue biopsy microarrays(BT/LL) and P-values (represent significance of differences in gene expression in T lep and L lep tissue biopsy microarrays) are displayed in the right two columns.
(TIF)
