# Supplementary figures and images for: Jagged1 Instructs Macrophage Differentiation in Leprosy
Source: PLoS Pathog. 2016 Aug 17;12(8):e1005808. doi: 10.1371/journal.ppat.1005808 (PMC4988718; doi:10.1371/journal.ppat.1005808)

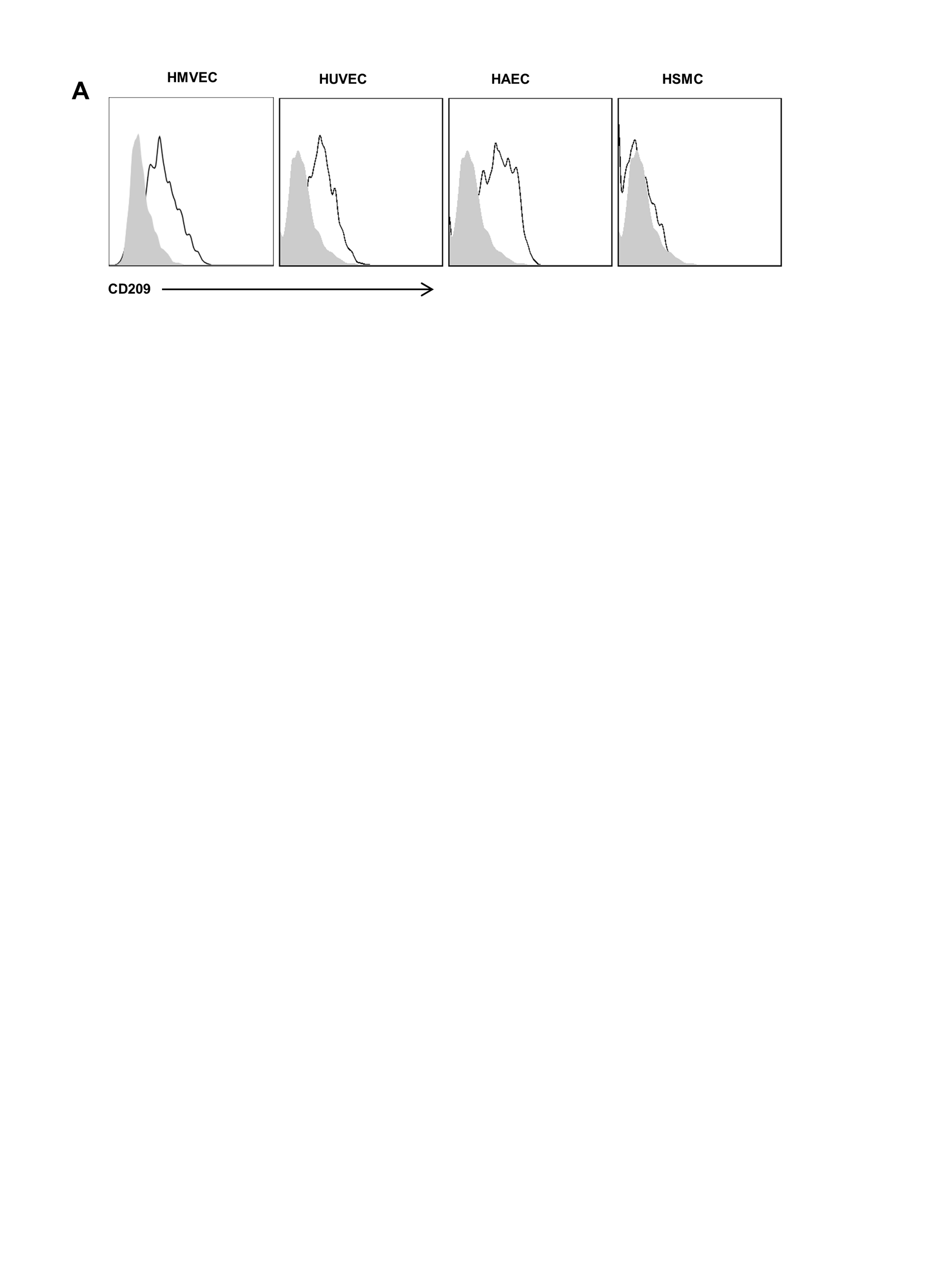

Supplement: S1 Fig — (A) Contact with vascular endothelium and not smooth muscle cells facilitates monocyte differentiation into CD209+MΦ. Histograms are representative of three independent experiments performed in triplicate. (TIF) [file ppat.1005808.s001.tif]

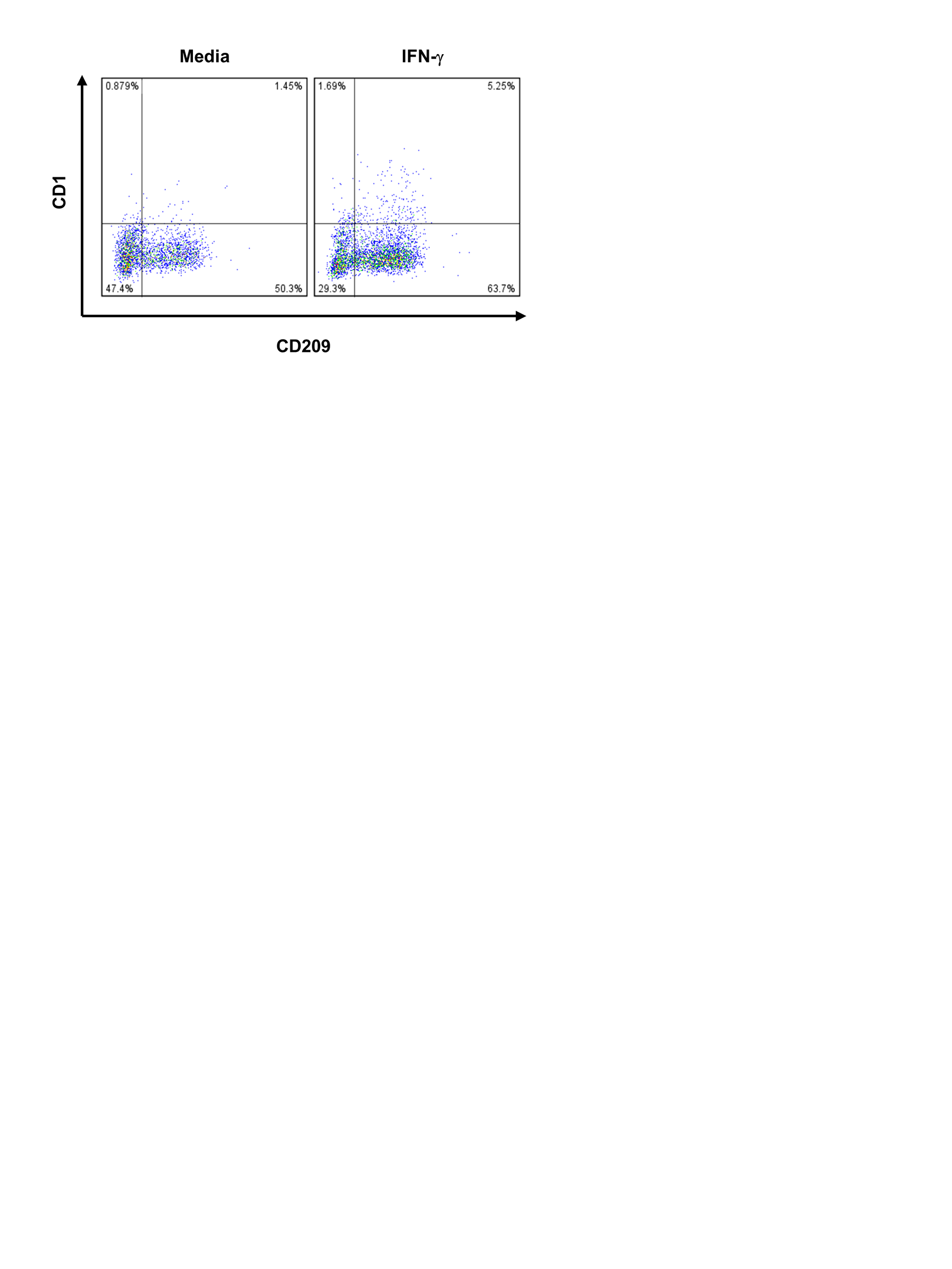

Supplement: S2 Fig — Log scale dot plots are representative of more than three independent donors performed in triplicate. (TIF) [file ppat.1005808.s002.tif]

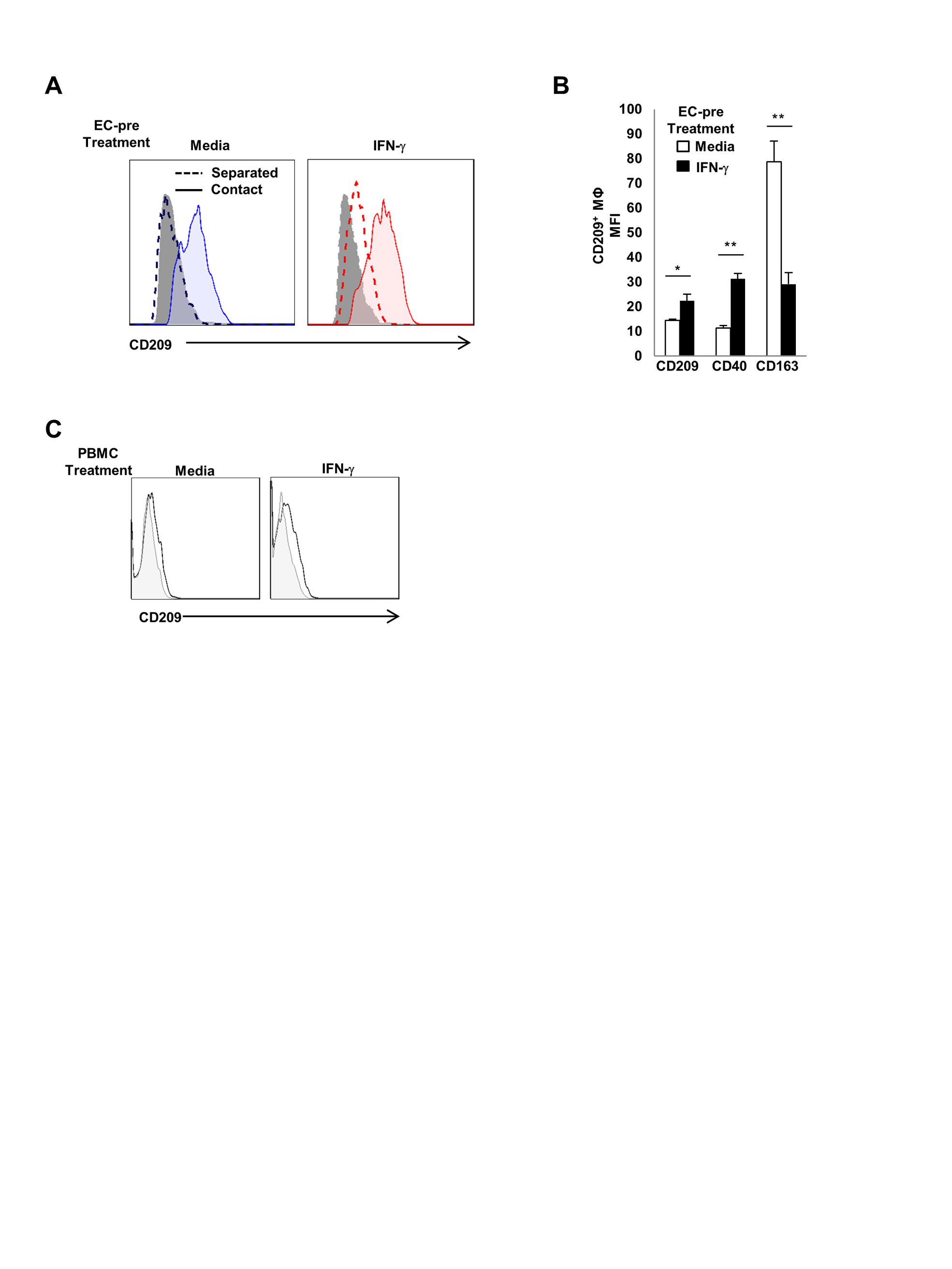

Supplement: S3 Fig — (A) Transwell experiments. EC were cultured in the upper chamber and treated with IFN-γ, with primary monocytes in the lower chamber. Histograms are representative of three independent donors performed in triplicate (B) IFN-γ treatment of HUVEC triggers purified CD14+ monocyte differentiation into antimicrobial CD209+MΦ. Data represent the mean +/- SEM from at least three independent donors (* p value < 0.05). (C) Treatment of PBMC with IFN-γ did not promote CD209+MΦ differentiation. Histograms are representative of more than three independent donors performed in triplicate. (TIF) [file ppat.1005808.s003.tif]

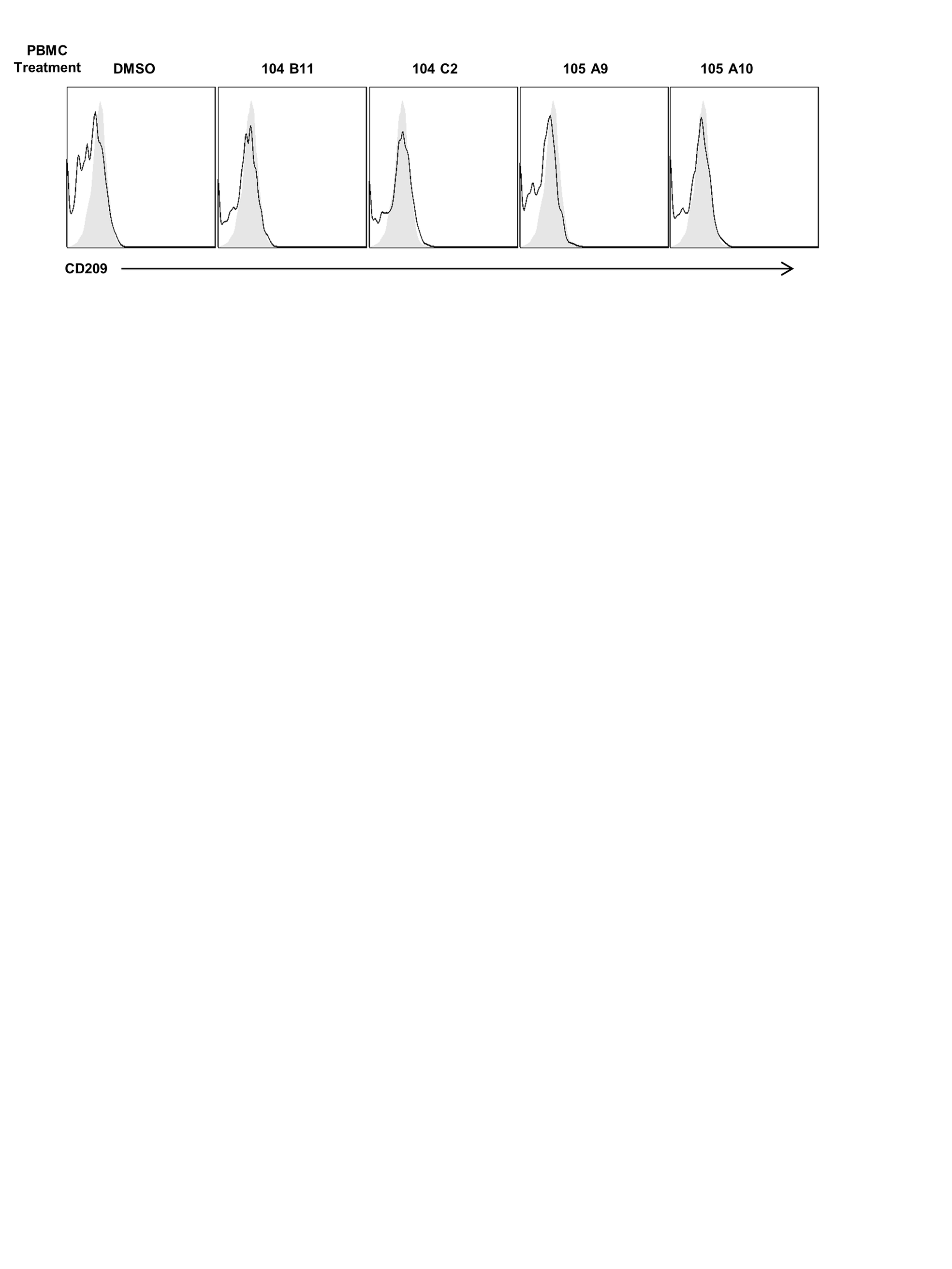

Supplement: S4 Fig — Stimulation of PBMC with active compounds (105 A9-A10, 104 B11-C2) did not result in the differentiation of CD209+MΦ. Histograms are representative of more than three independent donors performed in triplicate. (TIF) [file ppat.1005808.s004.tif]

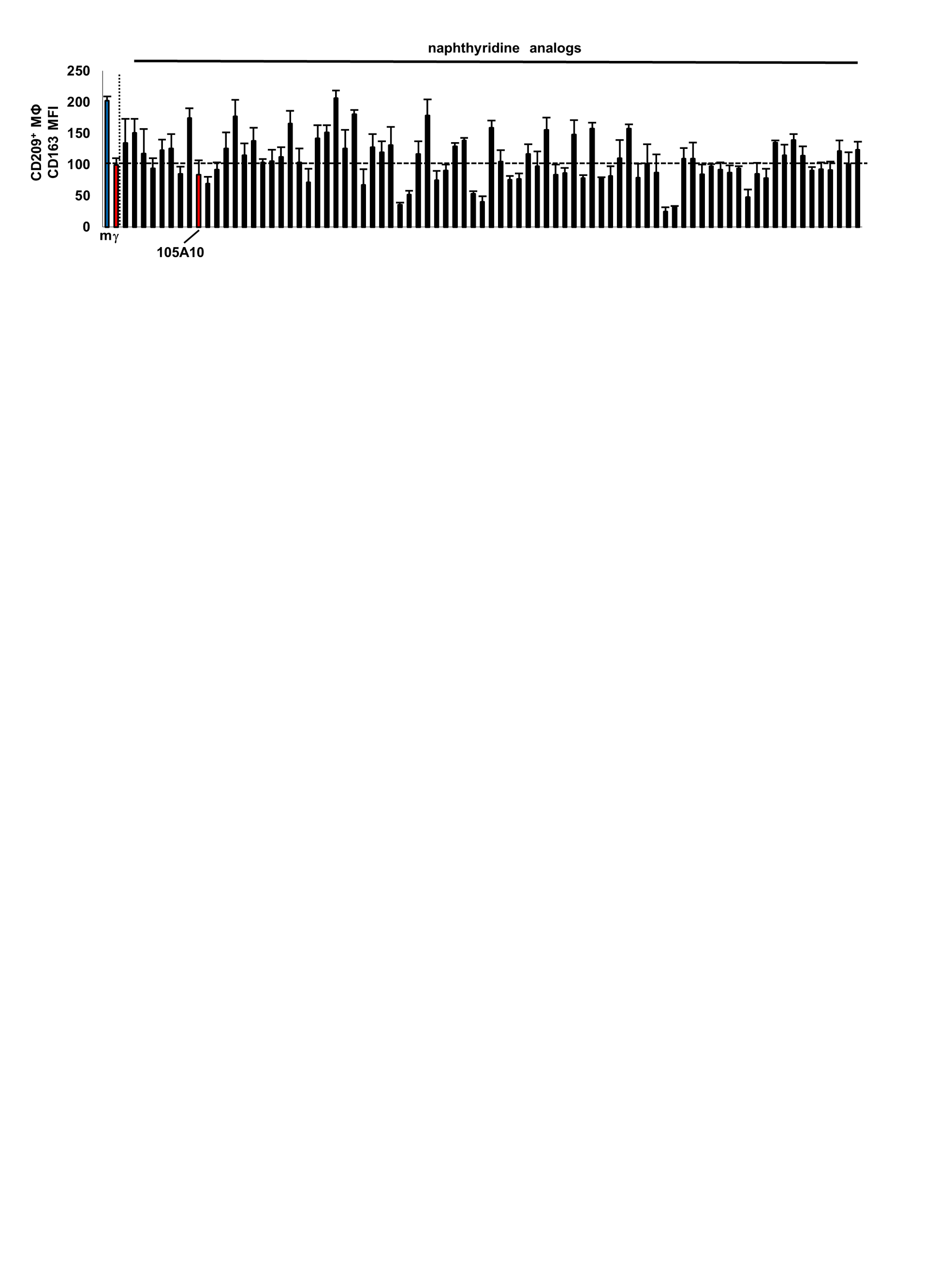

Supplement: S5 Fig — EC were treated with either media (m), IFN-γ (γ), 105A10, or naphthyridine analogs followed by co-culture with peripheral blood mononuclear cells. Data represent the mean +/- SEM from at least three independent donors. (TIF) [file ppat.1005808.s005.tif]

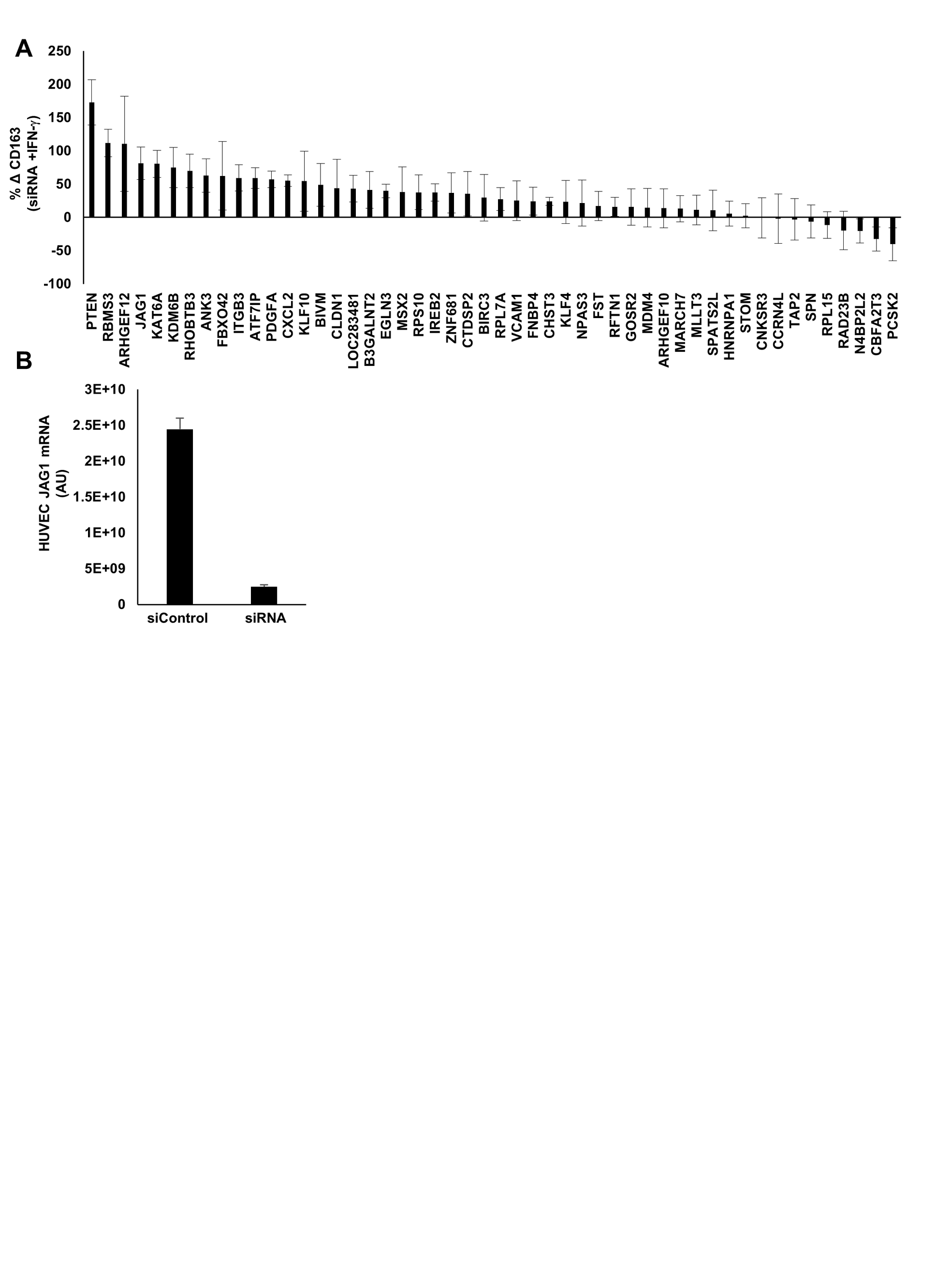

Supplement: S6 Fig — (A) Data represent the mean +/- SEM from five independent donors. (B) Validation of JAG1 mRNA knockdown by qPCR. Data represent the mean +/- SEM from at least three independent experiments. (TIF) [file ppat.1005808.s006.tif]

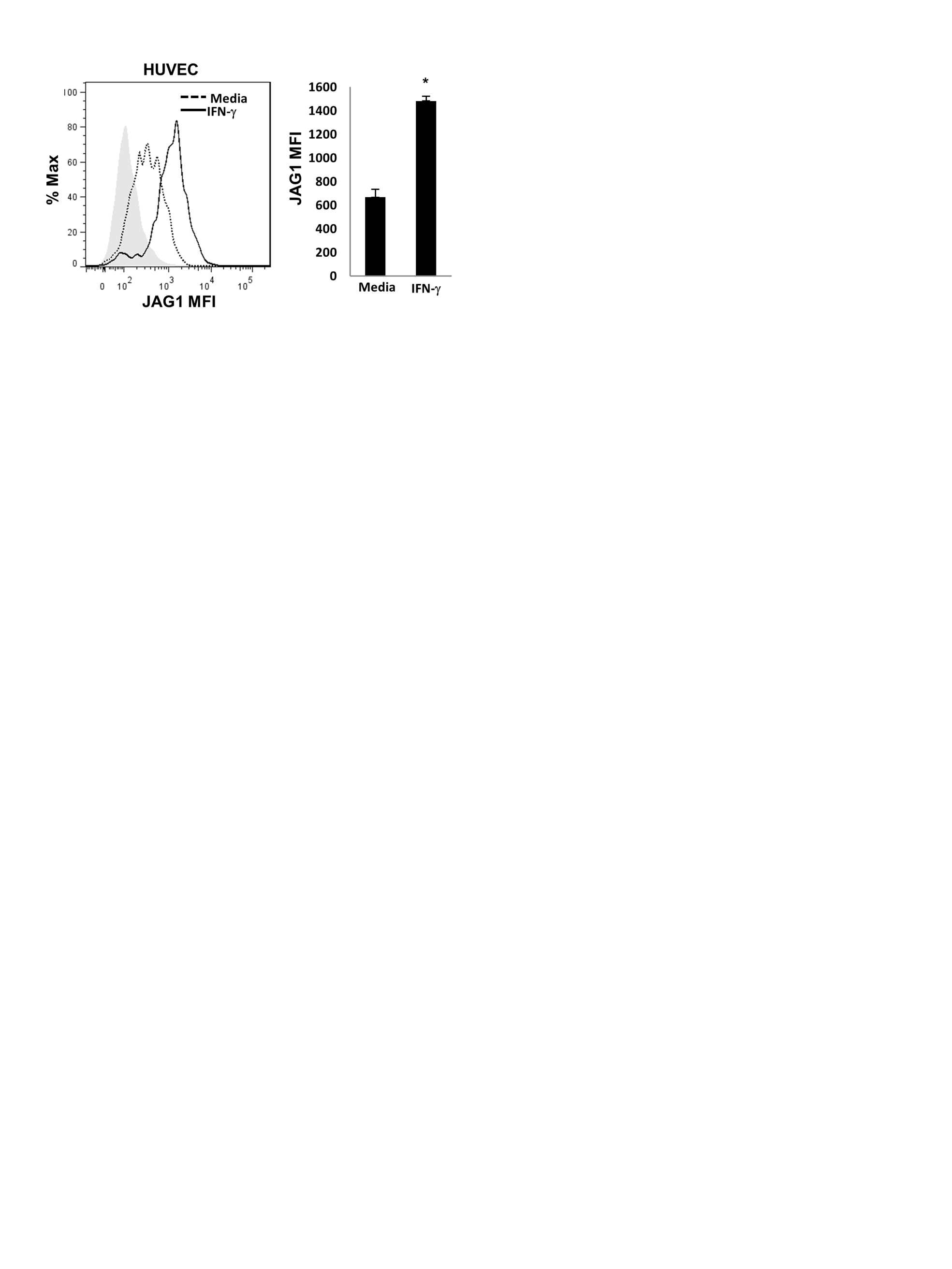

Supplement: S7 Fig — EC were cultured with or without IFN-γ and then assessed for JAG1 expression by flow cytometry. Data represent the mean +/- SEM from two independent experiments; filled histogram represents staining observed with isotype control. (TIF) [file ppat.1005808.s007.tif]

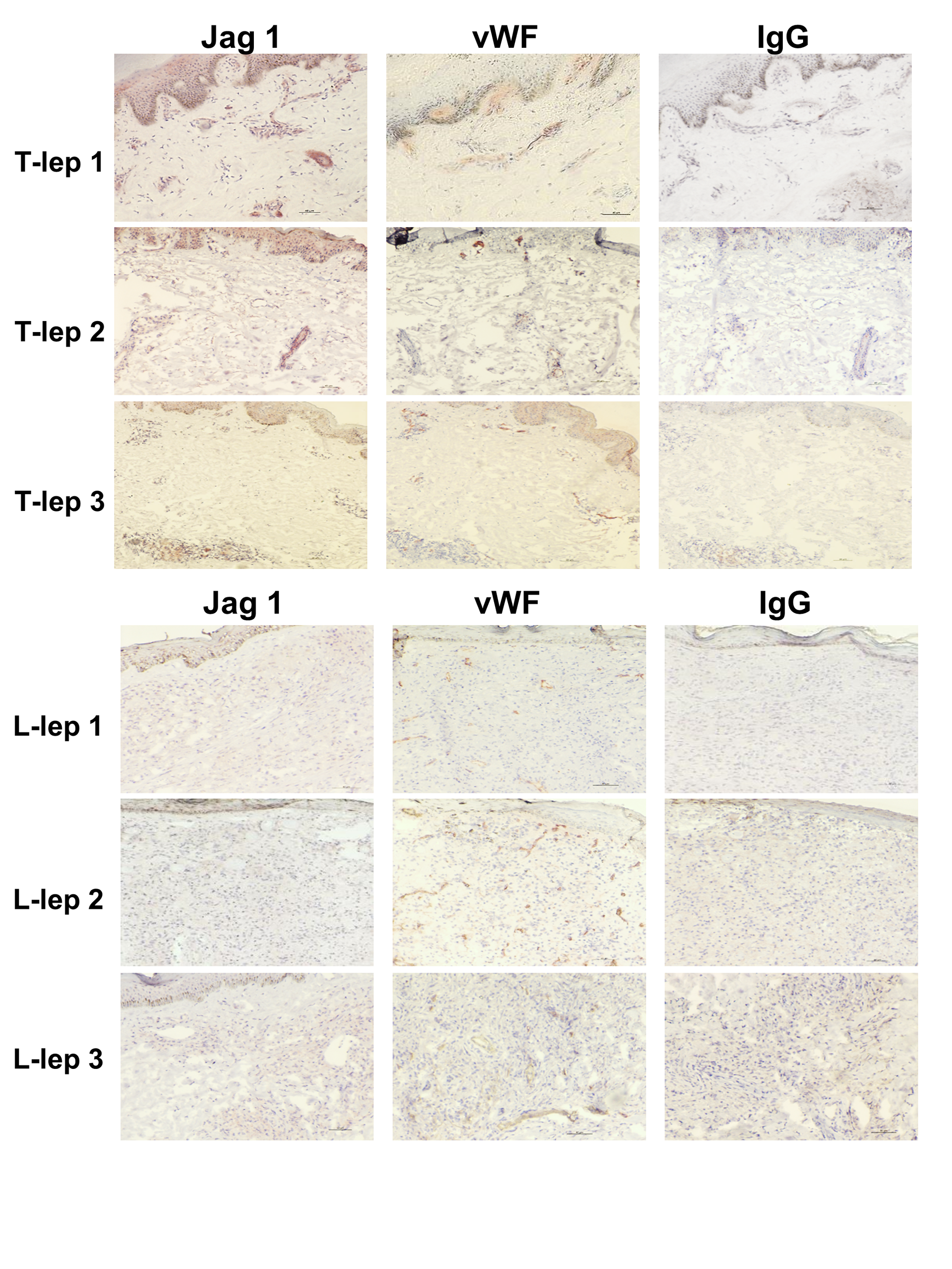

Supplement: S8 Fig — Immunohistochemistry (T-lep, n = 3 and L-lep, n = 3) staining of JAG1, vWF and IgG isotype controls. Positive staining of target proteins (JAG1 or vWF) is represented by red staining. (TIF) [file ppat.1005808.s008.tif]

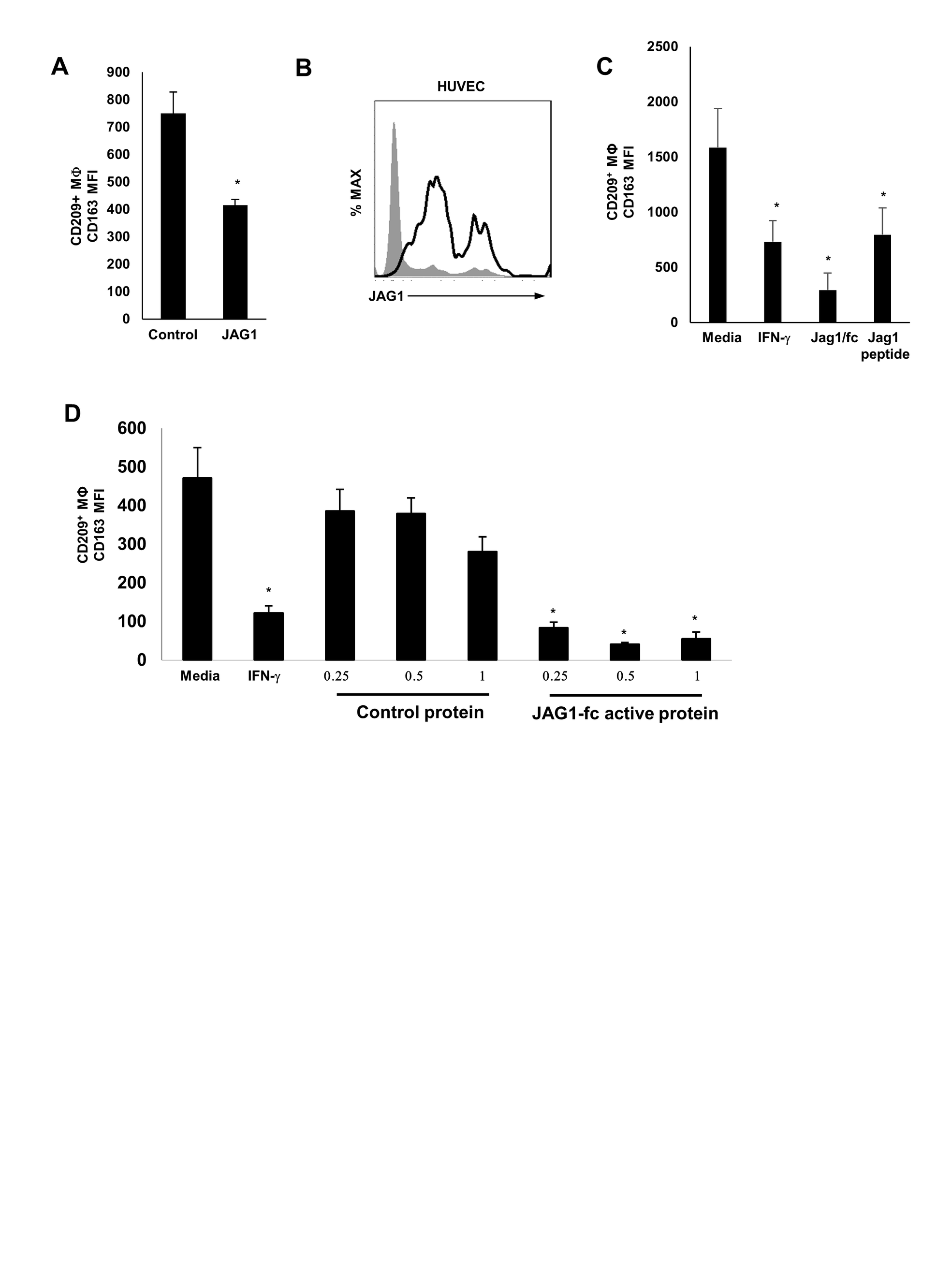

Supplement: S9 Fig — (A) Ectopic expression of JAG1 in transfected EC facilitates antimicrobial CD209+MΦ differentiation. Data represent the mean +/- SEM from at least three independent donors (* p value < 0.05) (B) Surface expression of JAG1 on M11-JAG1 transfected HUVEC. Histogram is representative of at least three independent experiments. (C) Addition of either JAG1 active peptide or soluble JAG1-fc protein to EC/PBMC co-culture facilitates CD209+CD163negMΦ differentiation. Data represent the mean +/- SEM from at least three independent donors. (* p value < 0.05). (D) Dose titration of soluble JAG1-fc active protein to EC/PBMC co-culture. Data represent the mean CD163MFI on CD209+ MΦ (+/- SEM from at least two independent donors. (* p value < 0.05)). (TIF) [file ppat.1005808.s009.tif]

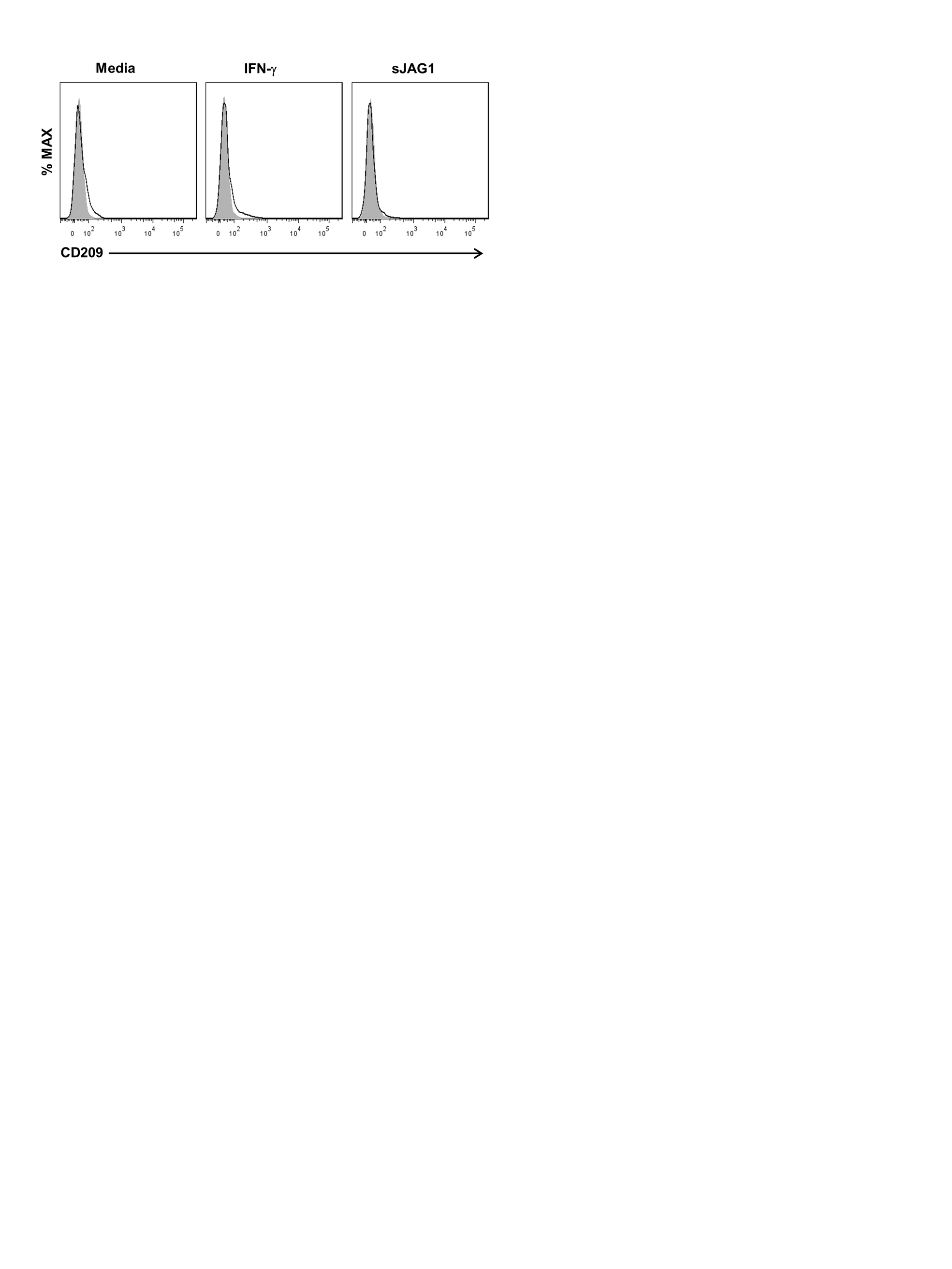

Supplement: S10 Fig — Direct stimulation of PBMC does not induce the differentiation of CD209+MΦ. Histograms are representative of more than three independent donors performed in triplicate. (TIF) [file ppat.1005808.s010.tif]

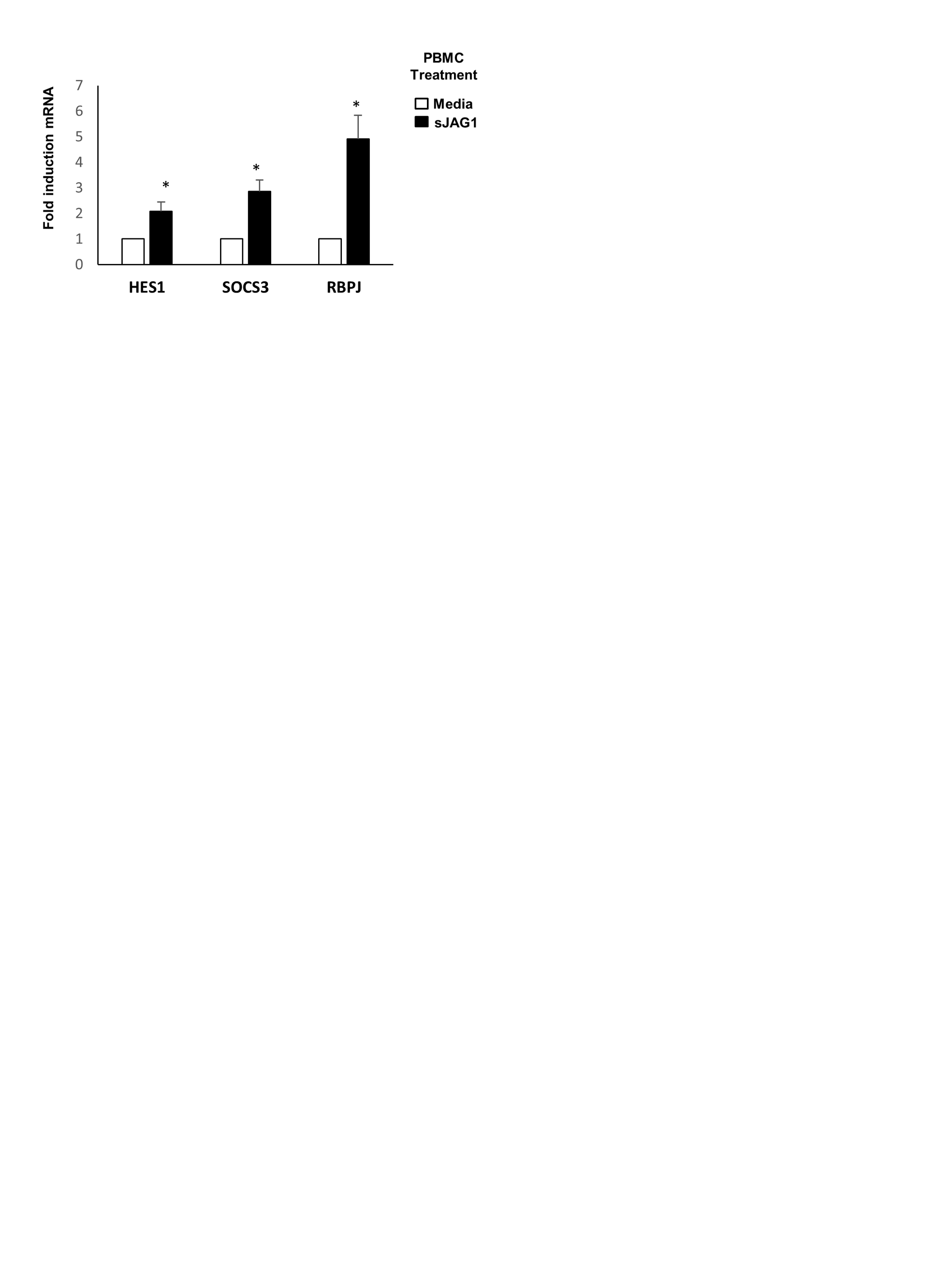

Supplement: S11 Fig — EC-monocyte co-cultures were treated with (black bars) or without (white bars) sJAG1 and after 2 days. NOTCH1 target gene expression was quantified by qPCR (p values: 0.02, 0.01, and 0.006 for HES1, SOCS3 and RBPJ, respectively). (TIF) [file ppat.1005808.s011.tif]

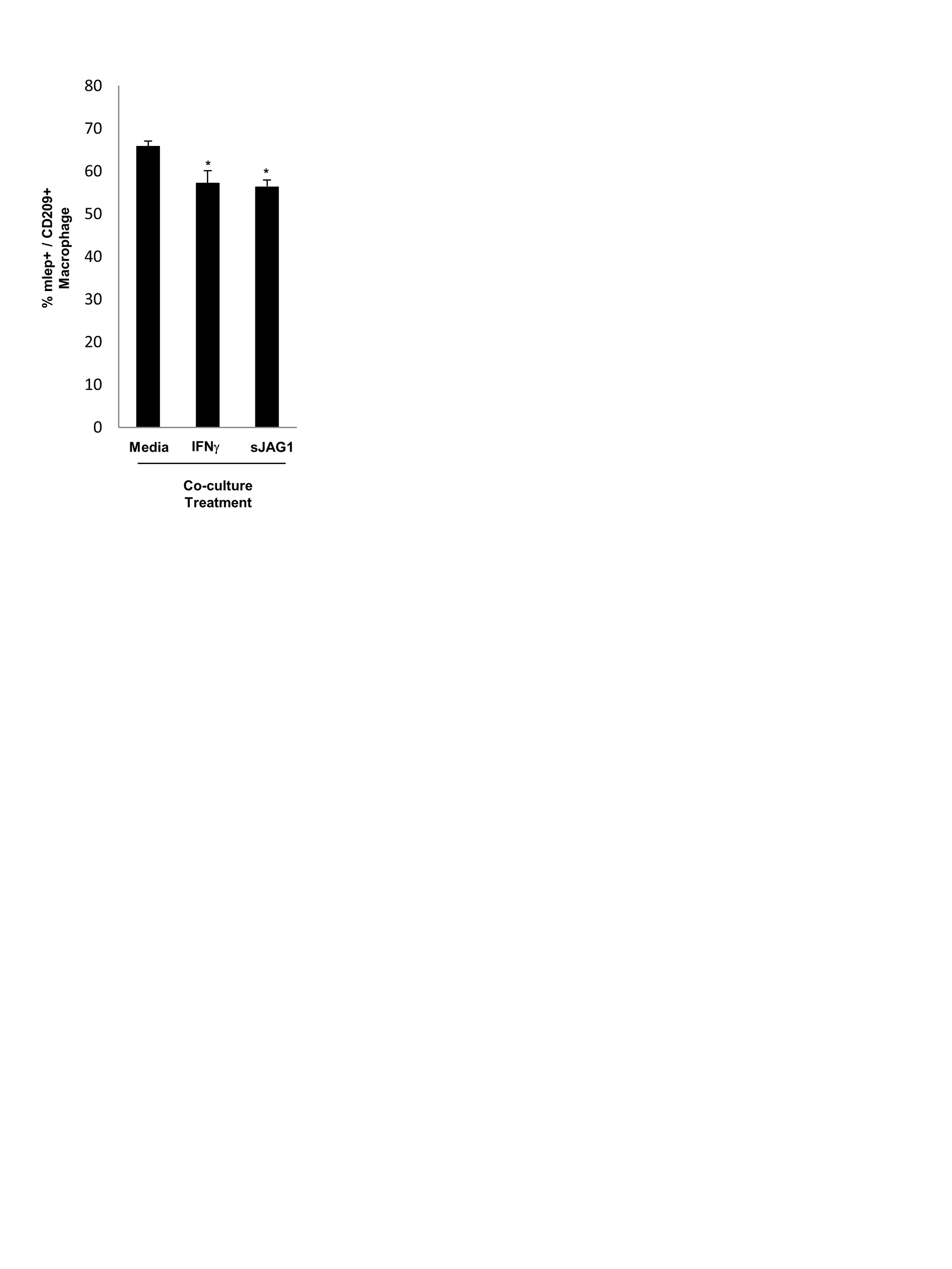

Supplement: S12 Fig — To determine uptake of M leprae during killing assays we used labeled M leprea (PE) and determined percent of CD209+ macrophages that had taken up M leprea by flow cytometry. Data represents three independent donors performed in triplicate. (* p value < 0.05). (TIF) [file ppat.1005808.s012.tif]

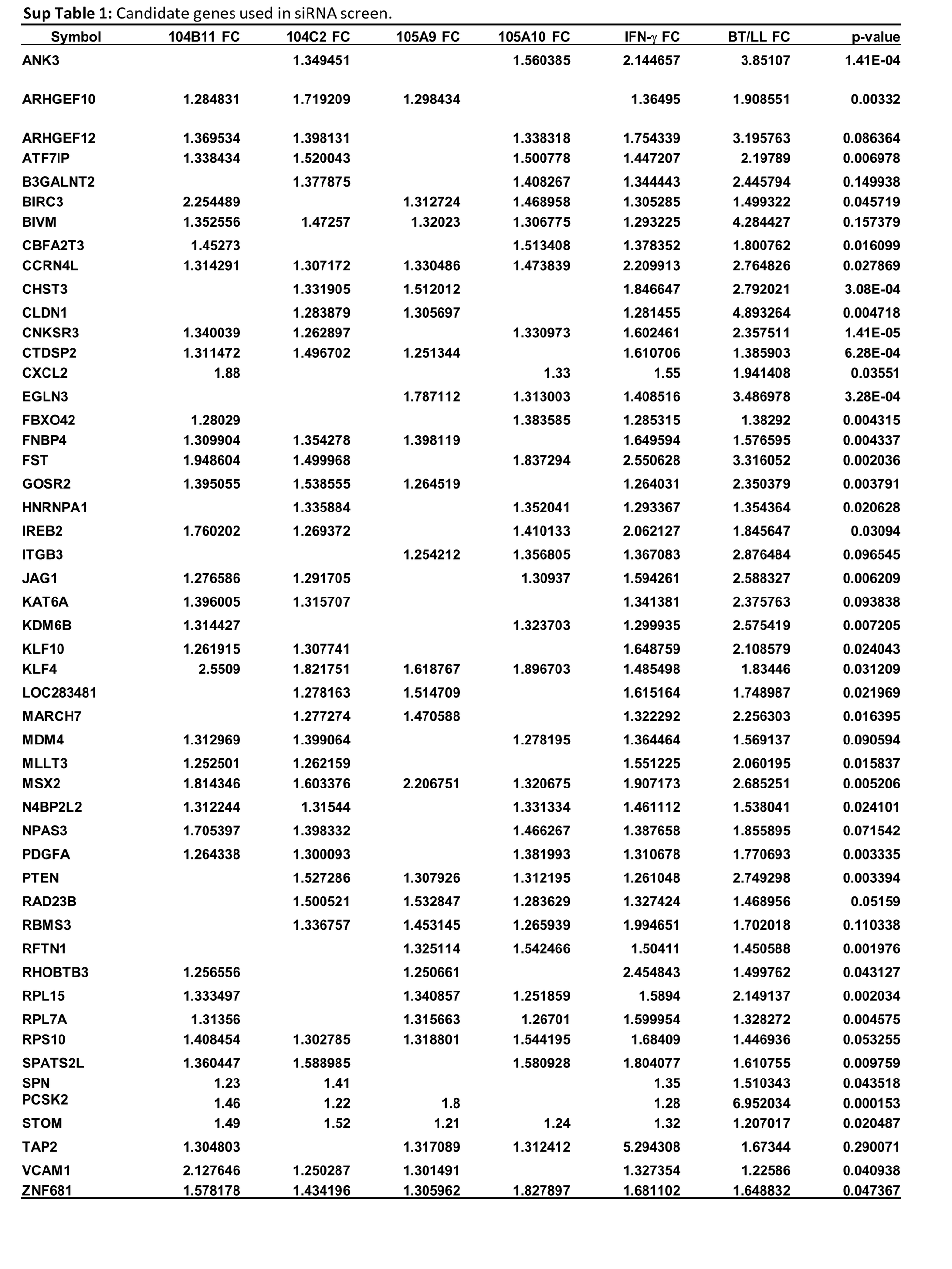

Supplement: S1 Table — Top fifty candidate genes used in siRNA screen. Fold change of gene expression in treated over media control HUVEC samples from microarray assays. In addition, the fold change of gene expression in T lep vs L lep tissue biopsy microarrays(BT/LL) and P-values (represent significance of differences in gene expression in T lep and L lep tissue biopsy microarrays) are displayed in the right two columns. (TIF) [file ppat.1005808.s013.tif]
